# Mobile and Web Apps for Weight Management in Overweight and Obese Adults: An Updated Umbrella Review and Meta-Analysis

**DOI:** 10.3390/ijerph22071152

**Published:** 2025-07-21

**Authors:** Felipe da Fonseca Silva Couto, Carlos Podalirio Borges de Almeida

**Affiliations:** Professional Master’s Program in Family Health (PROFSAÚDE), Institute of Health and Biological Studies, Federal University of the South and Southeast of Pará (UNIFESSPA), Avenida dos Ipês, s/n, Cidade Universitária, Loteamento Cidade Jardim, Marabá 68508-970, Pará, Brazil; carlos.almeida@unifesspa.edu.br

**Keywords:** obesity management, weight loss, mobile health, web-based interventions, eHealth, primary healthcare, public health

## Abstract

Obesity is a global epidemic with substantial health and economic impacts, making scalable weight management strategies essential. A comprehensive synthesis of eHealth interventions for weight management is needed to guide clinical practice. This umbrella review evaluated mobile and web-based interventions for weight loss in adults with overweight or obesity, compared to conventional or non-intervention controls. Systematic reviews were identified across five electronic databases from inception to February 2025. Two reviewers independently selected studies and assessed methodological quality using AMSTAR 2. Pooled estimates were calculated using random-effects models. Eleven systematic reviews (261 primary studies, 62,407 participants) were included. Mobile app interventions yielded a significant reduction in body weight (MD = −1.32 kg; I^2^ = 82%), as did long-term eHealth interventions (MD = −1.13 kg; I^2^ = 76%). Most meta-analyses showed high heterogeneity. Web-based interventions showed no significant effect. In conclusion, mobile apps and long-term eHealth interventions resulted in modest but statistically significant reductions in body weight, body mass index, and waist circumference. The evidence for web-based approaches remains inconclusive. Further research should focus on low-resource settings, primary care, and the integration of emerging technologies such as artificial intelligence. (PROSPERO CRD42025644218).

## 1. Introduction

Obesity and overweight are highly prevalent conditions that have reached epidemic proportions. In 2020, 2.2 billion adults were affected by overweight or obesity, with projections reaching 2.5 billion by 2025 and 3.3 billion by 2030 [1]. Overweight and obesity are significant risk factors for a wide range of noncommunicable diseases (NCDs), including cardiovascular disease, dyslipidemia, type 2 diabetes, certain cancers, and musculoskeletal and respiratory disorders [1]. Apart from its physical health consequences, obesity is closely linked to psychological disorders such as depression, anxiety, and eating disorders, significantly impacting the quality of life and life expectancy of these individuals [2,3].

These projections follow a concerning historical trend, with a significant increase in global deaths linked to high body mass index (BMI), which rose from 1.46 million in 1990 to 3.79 million in 2021 (a 153.97% increase). Similarly, Disability-Adjusted Life Years (DALYs) rose from 48.04 million to 128.52 million over the same period (a 167.57% increase) [4]. This burden has also translated into substantial economic costs. The World Obesity Federation (2024) projects an annual impact of USD 4.32 trillion by 2035, representing almost 3% of the global GDP [2]. For instance, Rocha et al. (2024) [5] reported that the Brazilian Health System (Sistema Único de Saúde [SUS]) spent USD 377.30 million on hospitalizations and high- and medium-complexity procedures to control NCDs attributable to high BMI in Brazil.

In response to this challenge, researchers and public health bodies have championed digital health technologies as scalable and promising strategies for weight management. The widespread use of smartphones and wearable devices has enabled mobile applications that support self-monitoring, behavior change, and personalized feedback [6,7]. Many of these tools incorporate behavioral models such as the Information-Motivation-Behavioral Skills (IMB) model to improve user adherence [8]. Despite these advantages, the evidence remains inconsistent regarding the effectiveness of digital interventions compared to conventional treatments (those without digital technologies) or no intervention [9,10]. Furthermore, as the COVID-19 pandemic significantly accelerated digital health adoption, much of the existing evidence predates this pivotal shift [11]. While telehealth has expanded rapidly, challenges such as limited access, digital literacy, and long-term adherence persist [11,12,13].

**Figure 1:**
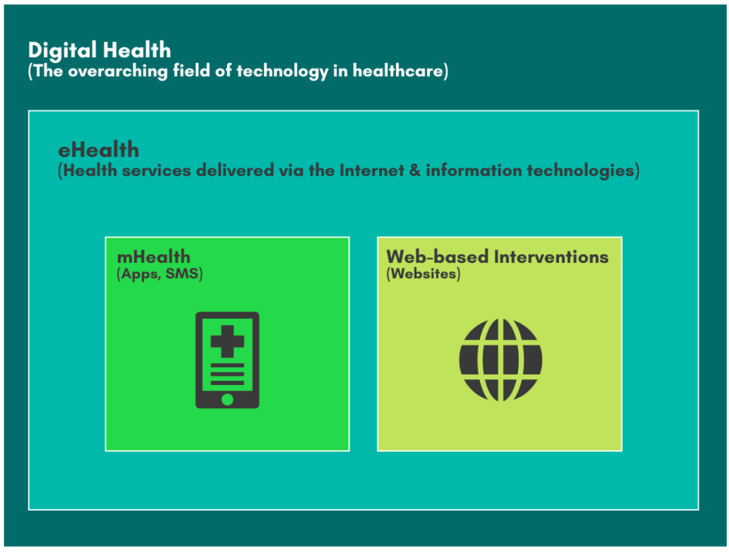
Conceptual framework of digital health terminology used in this review. The diagram illustrates the hierarchical relationship between digital health as the overarching field, eHealth as a major subfield, and mHealth and web-based interventions as the primary eHealth modalities analyzed.

To establish a clear conceptual framework, this review adopts the following terminology based on established definitions in the literature. Digital health is used as the broadest term, encompassing all applications of information and communication technologies in the health domain. A major subfield within this is electronic health (eHealth), which refers more specifically to the use of the internet and related technologies to deliver health information and services. Within the scope of this review, we analyze two primary modalities of eHealth: mobile health (mHealth), which involves interventions delivered specifically via mobile devices such as smartphones and tablets (e.g., applications, SMS messages), and web-based interventions, which are primarily delivered through websites. Figure 1 illustrates this hierarchical relationship. Throughout this paper, we use eHealth as a general term when discussing interventions collectively, and specify ‘mHealth’ or ‘web-based’ when referring to findings from these distinct modalities.

In the primary healthcare (PHC) context, digital health interventions, particularly mobile and web-based applications, hold significant promise. They offer accessible, scalable, and cost-effective solutions for weight management, potentially complementing traditional strategies used in the PHC context. Given the critical role of PHC in addressing obesity through preventive care and health promotion, robust evaluation of digital interventions is essential to inform international evidence-based practices and policies in diverse healthcare settings. Moreover, as Senbekov et al. (2020) [12] reported, digital health innovations require rigorous validation, especially in the absence of formal regulation.

Given this context, this study aims to synthesize and critically appraise the evidence on the effectiveness of mobile applications and web-based interventions for weight loss in overweight and obese adults, compared to conventional treatments without digital technologies or no intervention. Our findings suggest that mobile app and long-term eHealth interventions are effective for weight management in overweight and obese adults, while the effectiveness of web-based interventions remains inconclusive. Further research is needed in primary care and low-resource settings.

## 2. Materials and Methods

This umbrella review of systematic reviews adopted a meta-review strategy to evaluate the effectiveness of eHealth interventions, specifically mobile applications and web-based programs for weight loss in overweight and obese adults. In this study, eHealth interventions were defined as both mobile health (mHealth) applications (e.g., smartphone apps) and web-based programs, which were analyzed together when appropriate and separately in relevant subgroup analyses. This approach was chosen due to the multifactorial complexity of overweight and obesity and the need to consolidate dispersed and heterogeneous evidence, especially considering the lack of synthesis of post-COVID-19 studies.

The reporting of this meta-review followed the Preferred Reporting Items for Systematic Reviews and Meta-Analyses (PRISMA) guidelines [14] (Table S1) and was prospectively registered on PROSPERO (Centre for Reviews and Dissemination, University of York, York, UK) under the registration number CRD42025644218.

### 2.1. Eligibility Criteria

The review question was defined according to the PICOS framework:

1. Population: Adults (≥18 years) diagnosed with overweight or obesity (BMI ≥ 25).
2. Intervention: Use of mobile or web-based applications specifically designed as strategies for weight loss.
3. Comparator: Conventional treatment for overweight or obesity, including diet, exercise, behavioral therapy, pharmacological interventions, or minimal/no intervention, excluding digital technologies. Pharmacological interventions were considered an acceptable component of conventional treatment for comparator groups, as information about their use tends to be underreported or insufficiently detailed in systematic reviews. This a priori criterion was set to prevent the exclusion of potentially relevant reviews.
4. Outcomes: At least one of the following: reduction in BMI, waist circumference, body fat percentage, or overall weight loss.
5. Study design: Systematic reviews with meta-analyses, available in full text, assessing the effectiveness of mobile applications and web-based interventions compared to conventional treatments in adults with overweight or obesity. Systematic reviews including the general population were eligible if they performed separate analyses for adults and children. Exclusion criteria comprised clinical trials, abstracts, opinion pieces, narrative (non-systematic) reviews, and scoping reviews. No restrictions were applied regarding language or publication year at any stage of the search or screening process. However, all systematic reviews included in this umbrella review were published in English, and no potentially eligible reviews in other languages were retrieved. This a priori approach aligns with best practices for umbrella reviews and maximizes the comprehensiveness and temporal scope of the evidence, which is crucial in the rapidly evolving field of digital health.

Studies evaluating telemedicine interventions not involving mobile or web applications specifically designed for obesity management, interventions targeting conditions other than overweight/obesity, artificial intelligence-based apps or chatbots, and wearable device-based interventions (e.g., fitness trackers, smartwatches) were excluded.

### 2.2. Information Sources and Search Strategy

We performed electronic searches between 30 January and 7 February 2025, in five databases: Cochrane Library, Virtual Health Library, PubMed/MEDLINE, ScienceDirect, and Web of Science.

We developed the search strategy based on the PICOS framework using a combination of keywords derived from MeSH^®^ (Medical Subject Headings, National Library of Medicine, Bethesda, MD, USA) and DeCS® (Health Sciences Descriptors, BIREME, São Paulo, Brazil). However, searches were conducted using free-text terms in all databases. The terms were related to three key concepts: the population (“overweight”, “obesity”, “body weight”); the intervention (“mobile application”, “web-based intervention”, “mHealth”, “digital health”, “telehealth”); and the outcomes (“weight loss”, “body mass index”, “waist circumference”, “body fat”). Boolean operators (“AND” and “OR”) were used to combine these concepts and maximize search sensitivity.

As described above, no lower year limit was applied; all systematic reviews available in each database up to February 2025 were eligible for inclusion. Where possible, searches were limited to title and abstract fields, and publication type filters were applied to identify systematic reviews or meta-analyses.

The complete search strategy for each database is provided in File S1.

### 2.3. Study Selection and Data Extraction

All search results were exported and imported into the Rayyan platform (Qatar Computing Research Institute, Doha, Qatar) for screening. Rayyan’s automated tool was used to identify and remove duplicate records before title and abstract screening. Two independent reviewers (FFSC and CPBA) performed title/abstract and full-text screening. Disagreements were resolved through discussion to achieve consensus. Inter-rater agreement was assessed using Cohen’s Kappa coefficient, calculated in R (R Foundation for Statistical Computing, Vienna, Austria) (package ‘irr’). Reasons for full-text exclusion were documented and summarized. Final inclusion was determined by consensus following mutual review of extracted data (FFSC and CPBA).

### 2.4. Methodological Quality Assessment

The methodological quality of the included systematic reviews was assessed using the AMSTAR 2 tool [15], with both reviewers discussing and reaching a consensus on each assessment.

### 2.5. Assessment of Risk of Bias Across Studies

Publication bias was planned to be assessed in the included systematic reviews using funnel plot asymmetry and formal statistical tests (e.g., Egger’s or Begg’s test) when available. These assessments were extracted and summarized to evaluate the potential for cumulative reporting bias across studies.

### 2.6. Assessment of Overlap Among Primary Studies

To evaluate the degree of overlap among primary studies included in the systematic reviews, we constructed an overlap matrix and then calculated the Corrected Covered Area (CCA) according to the method proposed by Pieper et al. (2014) [16]. All lists of primary studies were standardized and merged, with duplicates removed to ensure an accurate count of unique studies.

### 2.7. Quantitative Synthesis and Subgroup Definitions

Meta-analyses were performed for all subgroups where clinical and methodological homogeneity allowed. Given the substantial heterogeneity in intervention types, comparators, outcomes, and study populations across the included systematic reviews, a single global meta-analysis pooling all interventions would not be methodologically appropriate. Instead, random-effects meta-analyses were conducted post hoc for clinically meaningful subgroups, including smartphone app interventions, web-based interventions (with and without human contact), and outcomes related to weight, BMI, waist circumference, and body fat percentage, as well as analyses stratified by intervention duration (short-term [≤6 months] and long-term [≥12 months]).

The principal summary measures were mean differences (MD) and standardized mean differences (SMD), both reported with 95% confidence intervals.

For each eligible meta-analysis, we systematically selected a single representative effect size to avoid double counting and ensure comparability. The following hierarchy of criteria was strictly applied in all cases:

1. Effect size corresponding to the longest follow-up period was selected whenever available;
2. If multiple effect sizes were available at the same follow-up, preference was given to estimates based on intention-to-treat (ITT) analysis;
3. In case of further ties, the result with the largest sample size was chosen.

This approach was applied systematically across all included reviews and subgroups, ensuring methodological consistency and minimizing selection bias. Where at least two eligible effect sizes were available, quantitative synthesis was performed; single-study estimates were described narratively. All meta-analyses were conducted in R (version 4.4.3, R Foundation for Statistical Computing, Vienna, Austria) using the ‘meta’ package. Heterogeneity was assessed using the I^2^ statistic for each meta-analysis.

For each subgroup, effect sizes were filtered and harmonized using regular expressions and explicit inclusion/exclusion logic to match clinical definitions (e.g., excluding SMS/telephone from smartphone app analyses).

## 3. Results

### 3.1. Study Selection

We retrieved 281 records from five databases. After removing 58 confirmed duplicates, 223 unique records remained for title and abstract screening. Inter-rater agreement at this stage was substantial (κ = 0.805), with 195 records excluded and 28 full-text articles assessed for eligibility. The full-text review yielded almost perfect agreement between reviewers (κ = 0.928). The code used to assess inter-rater agreement is available in File S2.

**Figure 2:**
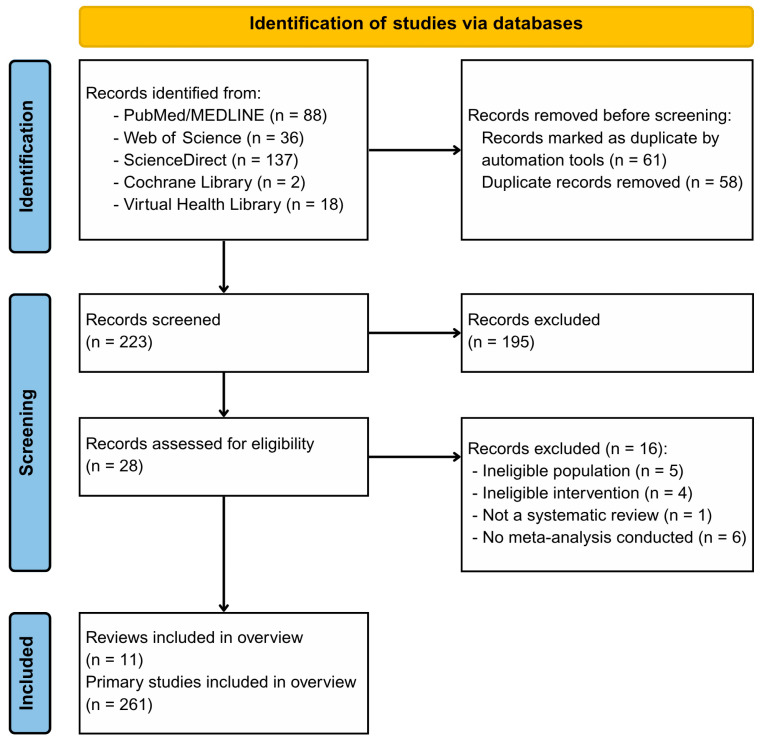
PRISMA Flow diagram for identification of systematic reviews and primary studies. Note: One review was excluded post-screening, as it was identified as a conference abstract without primary study data, resulting in a final sample of 11 reviews.

After consensus, 12 systematic reviews were selected for data extraction. However, one study (Li et al., 2024) [17] was excluded due to the absence of reported primary study data. As this information was not apparent from the publication, the authors were contacted for clarification and confirmed that the paper was a conference abstract presenting only partial results, with no access to primary study data. Consequently, the final sample comprised 11 systematic reviews. Collectively, these reviews synthesized data from 261 primary studies, encompassing a total of 62,407 participants. The subsequent analyses on geographical distribution and income level are based on the subset of these studies for which country data were reported. The study selection process is summarized in Figure 2, and the reasons for full-text exclusions are listed in Table S2.

### 3.2. Study Characteristics

The 11 systematic reviews [9,10,18,19,20,21,22,23,24,25,26], were published between 2010 and 2024 and covered a broad range of digital health interventions for weight management in adults with overweight or obesity. On average, each review included 23.7 primary studies (SD = 21.8, median = 18, range 6–84) and a mean sample size of 5673 participants (SD = 6159, median = 2630, range 632–24,010).

Most reviews focused on adults, although a minority also included individuals at risk for metabolic disease [18,23] or with prediabetes [25]. The most common interventions in the meta-analyses were mobile health (mHealth), such as smartphone apps, SMS, and wearables [10,18,19,20,22,23]. These were followed by web-based digital interventions [9,21,24,26] and a single mixed intervention including both web and mobile components [25]. In these studies, comparison groups most frequently received usual care, were placed on a wait-list, or had a minimal intervention. The intervention duration was typically short-term, with a median of 6 months (range 3–24 months).

**Figure 3:**
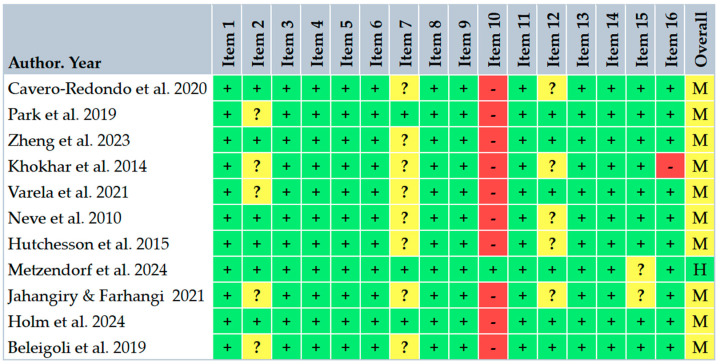
Critical appraisal of included reviews according to AMSTAR 2 criteria [15]. Green (+) = yes; yellow (?) = partial yes; red (−) = no; (H) = high confidence; (M) = moderate confidence [9,10,18,19,20,21,22,23,24,25,26].

The primary outcome reported across all reviews was the change in body weight (kg), expressed as mean difference (MD) or standardized mean difference (SMD). Secondary outcomes included BMI, waist circumference, body fat percentage, and adherence or dropout rates. Risk of bias and methodological quality were variably assessed; all reviews reported at least moderate quality by AMSTAR 2 criteria (Figure 3).

**Figure 4:**
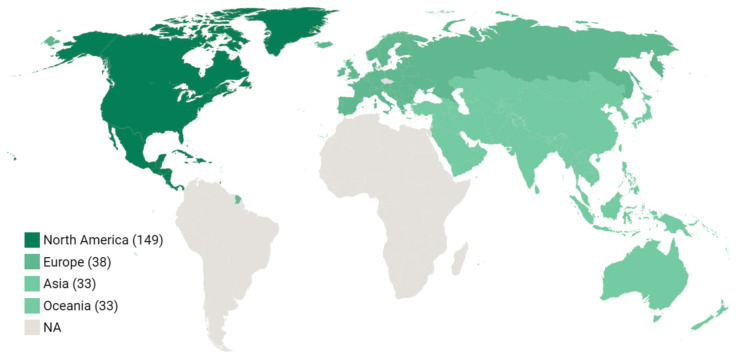
Regional distribution of primary studies.

Among the 254 primary studies for which country or region was reported, 149 (58.7%) were from North America, 38 (15.0%) from Europe, 33 (13.0%) from Oceania, 33 (13.0%) from Asia, and 1 study (0.4%) was multisite. Figure 4 depicts the capture areas of these primary studies.

Regarding country income classification, of the 254 studies with available data, the vast majority, 237 studies (93.3%), were conducted in high-income countries. Additionally, seven studies (2.8%) took place in upper-middle-income countries and seven studies (2.8%) in lower-middle-income countries. The multisite study (0.4%) and two studies from Asia without a specified country (0.8%) were not classified by income level.

The primary studies included across reviews were published between 1999 and 2024, with a median publication year of 2013 and the majority published after 2010. Notably, three systematic reviews included primary studies published during or after the COVID-19 pandemic (2020 or later), reflecting the recent evolution in eHealth interventions. A summary of included reviews and their main characteristics are provided in Table 1.

### 3.3. Overlap Among Primary Studies

After deduplication and standardization, 212 unique primary studies were identified across the 11 included systematic reviews out of a total of 261 initial study inclusions. This reduction from the nominal total reflects substantial overlap among reviews: many of the same primary studies were included in more than one systematic review, sometimes under minor citation variations or reported as separate entries for different analyses or follow-ups. This rigorous harmonization prevents artificial inflation of the evidence base, as recommended in the methodology for umbrella reviews. The resulting Corrected Covered Area (CCA) was 2.3%, indicating slight overlap according to established thresholds [16]. The low degree of overlap suggests minimal risk of evidence inflation or redundancy in our umbrella review. Further details and the complete overlap matrix are provided in Table S3.

### 3.4. Risk of Bias Within Studies

Among the 11 included systematic reviews, the most frequently used tool for risk of bias assessment was the Cochrane Risk of Bias tool (original or RoB 2), applied in 7 reviews [10,18,19,21,23,25,26]. The Jadad scale was used in two reviews [20,24], and the Joanna Briggs Institute instrument (JBI-MAStARI) was used in two reviews [9,22]. One review [18] additionally used the ROBINS-I tool for non-randomized studies. One review [26] assessed the risk of bias using GRADE domains rather than a standalone tool. All reviews reported some method of risk of bias assessment for the included primary studies.

### 3.5. Risk of Bias Across Studies

Of the eleven included systematic reviews, eight (73%) formally evaluated publication bias in their meta-analyses using funnel plot inspection and/or statistical tests such as Egger’s or Begg’s test [10,18,19,20,21,22,24,25]. Among these, seven reviews (64%) reported no evidence of publication bias for their primary outcomes, whereas one review [18] identified significant publication bias using Egger’s test and funnel plot asymmetry. Three reviews [9,23,26] did not report a formal assessment of publication bias.

### 3.6. Representative Effect Sizes from Individual Studies

A total of 26 effect sizes were extracted from the eligible meta-analyses included in this umbrella review. Of these, four were standardized effect sizes (Cohen’s d, standardized mean difference [SMD]), fifteen were unstandardized effect sizes (mean difference in kilograms, BMI in kg/m^2^, risk ratio [RR]), and seven were classified as other effect measures, including odds ratios, proportions, or effect sizes not clearly defined as standardized or unstandardized. These results cover main outcomes such as weight loss, weight change, BMI change, and waist circumference.

Table 2 presents these effect sizes together with their 95% confidence intervals (CIs), PICO categories, heterogeneity (I^2^), publication bias, and strength of evidence according to the umbrella review methodology. When available, the GRADE rating for certainty of evidence is also included.

The directions of both standardized and unstandardized effect sizes are presented as originally reported. The number of *p*-values for representative effect sizes is also provided in Table 2.

### 3.7. Moderators

We summarized the results from moderator analyses conducted across the included meta-analyses (full details available in Table 3). Out of the 11 meta-analyses included, 10 (91%) reported conducting some form of moderator analysis. Most examined mobile health (mHealth) interventions (e.g., smartphone apps, SMS, wearables) [10,18,22,23], web-based digital interventions [9,21,24,26], or eHealth lifestyle interventions that included both web-based and mobile components [25]. Commonly tested moderators included sample characteristics (such as age, sex, baseline BMI, and region) and intervention features (such as comparator type, platform/device, intervention duration, presence of behavioral counseling, feedback frequency, and digital contact intensity).

Among the ten reviews reporting moderator analyses, two meta-analyses (18%) conducted formal meta-regression to statistically assess the contribution of moderators to heterogeneity [18,26]. In both cases, intervention type, comparator group, and intervention duration were identified as significant moderators influencing weight loss outcomes.

The remaining eight meta-analyses (73%) conducted only subgroup analyses without formal meta-regression modeling. For instance, Khokhar et al. (2014) [20] stratified results by device type (mobile phone vs. PDA), while others evaluated intervention duration, feedback mechanisms, geographical region, or participant characteristics.

Across studies, frequently reported significant moderators included shorter intervention duration (<6 months), smartphone-based delivery, inclusion of human contact or behavioral counseling, and trials conducted in specific regions (particularly North America and Asia). However, these effects were not always consistent; in some cases, moderator effects were limited to specific subgroups or were not sustained at longer follow-up durations or when compared to active control groups.

### 3.8. Quantitative Synthesis and Subgroup Meta-Analyses

Meta-analyses were conducted for each clinically and methodologically homogeneous subgroup with sufficient data. The complete code used for data preprocessing, selection of representative effect sizes, and statistical analyses is available in File S3.

Results for smartphone app interventions, web-based interventions (with and without human contact), and outcomes related to weight, BMI, waist circumference, and body fat, as well as analyses stratified by intervention duration (short-term and long-term), are summarized below, following best practices for umbrella reviews in this field [27,28].

#### 3.8.1. Mobile App Interventions

**Figure 5:**
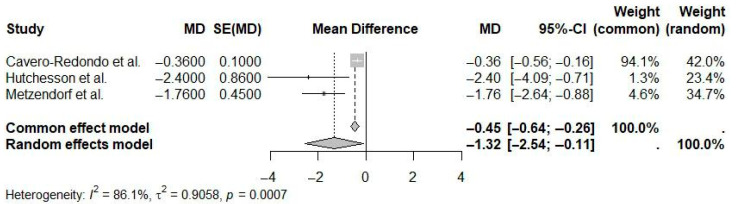
Pooled effect of mobile app interventions versus usual care on weight loss in overweight and obese adults (random-effects meta-analysis). The diamond represents the overall pooled mean difference and its 95% confidence interval. The vertical dotted line indicates the line of no effect (mean difference = 0) [18,22,23].

A random-effects meta-analysis pooling studies evaluating mobile application interventions exclusively (i.e., smartphone apps, excluding SMS and telephone-based technologies) demonstrated a statistically significant pooled mean difference in weight loss of −1.32 kg (95% CI: −2.54 to −0.11; I^2^ = 86.1%) compared to usual care or minimal intervention (Figure 5). The significant heterogeneity observed indicates considerable variability across studies regarding app features, target populations, and intervention designs, suggesting caution in generalizing these results.

#### 3.8.2. Web-Based Interventions with Human Contact

**Figure 6:**
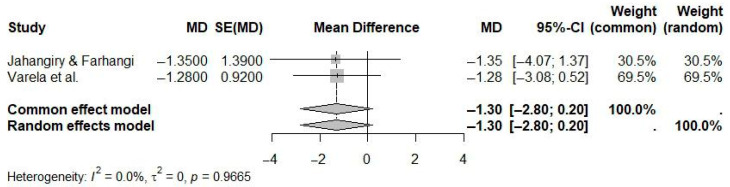
Pooled effect of web-based interventions with human contact versus controls on weight loss (random-effects meta-analysis). See the legend of Figure 5 for explanation of symbols and lines [21,24].

In analyses restricted to web-based interventions incorporating human contact (e.g., coaching or support), the pooled mean difference in weight loss was −1.30 kg (95% CI: −2.80 to 0.20; I^2^ = 0.0%) compared to controls (Figure 6). Although this effect was not statistically significant, the direction favored interventions with human support. The absence of observed heterogeneity suggests high consistency among the included studies.

#### 3.8.3. Web-Based Interventions Without Human Contact

**Figure 7:**
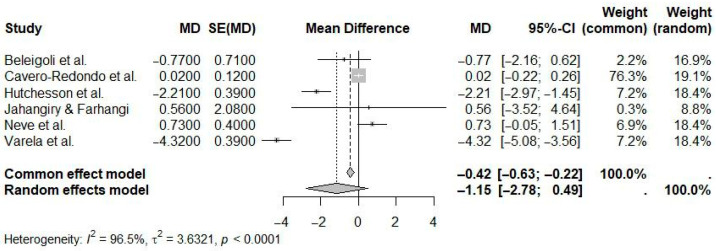
Pooled effect of web-based interventions without human contact versus controls on weight loss (random-effects meta-analysis). See the legend of Figure 5 for explanation of symbols and lines [9,18,21,22,24,26].

For web-based interventions without human contact, the pooled mean difference in weight loss was −1.15 kg (95% CI: −2.78 to 0.49; I^2^ = 96.5%) (Figure 7). This effect was not statistically significant, and the very high heterogeneity highlights substantial variability among the intervention characteristics and study populations.

#### 3.8.4. Weight Outcome

**Figure 8:**
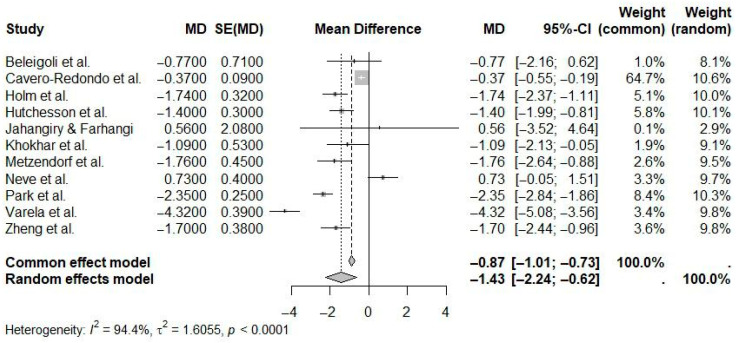
Pooled effect of eHealth interventions versus controls on weight loss (random-effects meta-analysis). See the legend of Figure 5 for explanation of symbols and lines [9,10,18,19,20,21,22,23,24,25,26].

A random-effects meta-analysis of studies reporting weight outcomes demonstrated a statistically significant pooled mean difference of −1.43 kg (95% CI: −2.24 to −0.62; I^2^ = 94.4%) favoring eHealth interventions compared to controls (Figure 8). Although statistically significant, the very high heterogeneity underscores considerable variation in intervention methods and study populations.

#### 3.8.5. BMI Outcome

**Figure 9:**
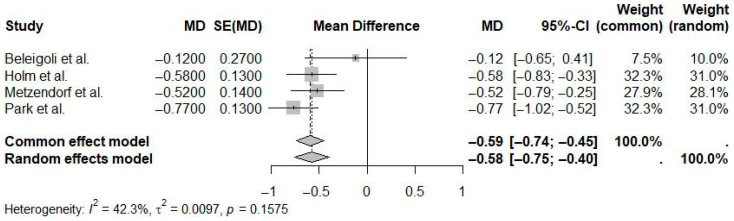
Pooled effect of eHealth interventions versus controls on BMI reduction (random-effects meta-analysis). See the legend of Figure 5 for explanation of symbols and lines [10,23,25,26].

Studies assessing BMI outcomes yielded a statistically significant pooled mean difference of −0.58 kg/m^2^ (95% CI: −0.75 to −0.40; I^2^ = 42.3%), favoring eHealth interventions compared to controls (Figure 9). The moderate heterogeneity suggests greater consistency in BMI outcomes compared to weight outcomes.

#### 3.8.6. Waist Circumference Outcome

**Figure 10:**
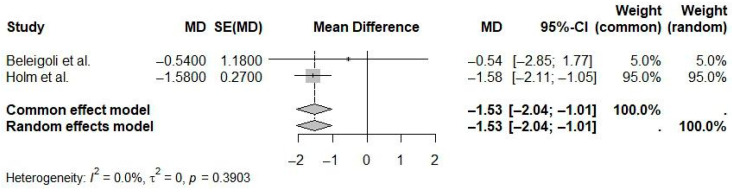
Pooled effect of eHealth interventions versus controls on waist circumference reduction (random-effects meta-analysis). See the legend of Figure 5 for explanation of symbols and lines [25,26].

The pooled mean difference for waist circumference was statistically significant at −1.53 cm (95% CI: −2.04 to −1.01; I^2^ = 0.0%), favoring eHealth interventions (Figure 10). No heterogeneity was observed, indicating high consistency among the results of the included studies.

#### 3.8.7. Short-Term eHealth Interventions (≤6 Months)

**Figure 11:**
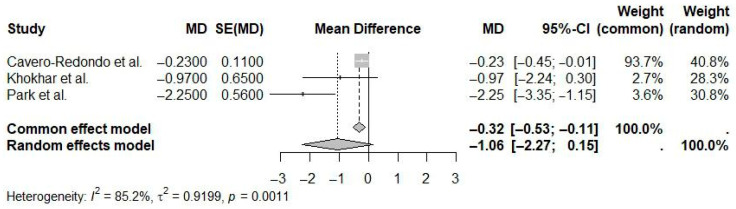
Pooled effect of short-term (≤6 Months) eHealth interventions versus controls on weight loss (random-effects meta-analysis). See the legend of Figure 5 for explanation of symbols and lines [10,18,20].

A random-effects meta-analysis evaluating short-term eHealth interventions (≤6 months) indicated a pooled mean difference in weight loss of −1.06 kg (95% CI: −2.27 to 0.15; I^2^ = 85.2%) compared to controls (Figure 11). This effect was not statistically significant, with high heterogeneity suggesting significant variability in interventions and study populations.

#### 3.8.8. Long-Term eHealth Interventions (≥12 Months)

**Figure 12:**
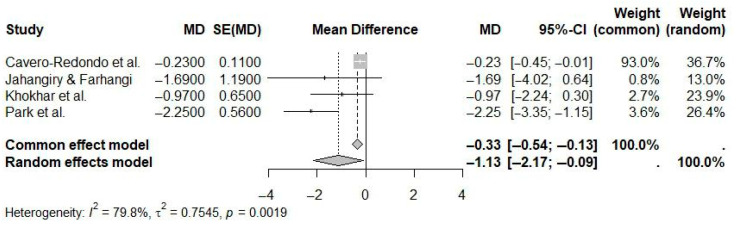
Pooled effect of long-term (≥12 Months) eHealth interventions versus controls on weight loss (random-effects meta-analysis). See the legend of Figure 5 for explanation of symbols and lines [10,18,20,24].

For long-term eHealth interventions (≥12 months), the pooled mean difference in weight loss was statistically significant at −1.13 kg (95% CI: −2.17 to −0.09; I^2^ = 79.8%) compared to controls (Figure 12). The moderate to high heterogeneity highlights notable variability in intervention characteristics and populations across studies.

**Figure 13:**
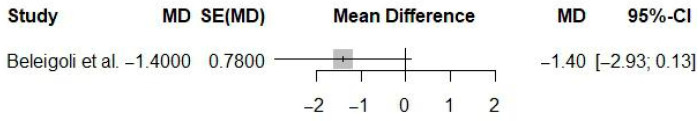
Pooled mean difference in body fat percentage for eHealth interventions compared to controls (single-review estimate). See the legend of Figure 5 for explanation of symbols and lines [26].

Additional subgroup analysis was performed for body fat percentage outcomes. However, this outcome was derived from a single systematic review, precluding umbrella meta-analysis. The reported effect size (MD = −1.40%, 95% CI: −2.93 to 0.13) (Figure 13) represents the pooled estimate from this single review.

### 3.9. Qualitative Synthesis

Qualitative findings indicate that mHealth interventions, particularly those emphasizing lifestyle self-monitoring, effectively enhance autonomy, self-management, and user empowerment, thereby increasing adherence rates compared to traditional methods [18]. Smartphones, due to their widespread accessibility, ease of use, and ability to reduce clinical burden by decreasing the frequency of in-person contacts, emerged as pivotal technological tools in this context [18].

Despite encouraging short-term results, the long-term sustainability of mHealth interventions remains uncertain. Qualitative synthesis from the included systematic reviews suggested that the primary role of mHealth interventions may be more appropriate as initial catalysts rather than stand-alone solutions for long-term weight maintenance [10,20]. Holistic and integrated mHealth programs, which comprehensively address nutrition, physical activity, and mental health, have demonstrated multifaceted benefits; however, sustained engagement tends to diminish without sufficient human interaction [19].

Web-based interventions notably depend on frequent, personalized feedback from healthcare professionals, highlighting human interaction as essential to enhance user motivation, adherence, and safety [9,21]. Additionally, structured behavioral components, goal-setting, and consistent self-monitoring significantly enhance the effectiveness of these interventions [22,24,25]. Despite initial successes, web-based programs often face challenges in sustaining long-term weight loss, underscoring the necessity for continuous user engagement and strategically designed digital features to preserve achieved outcomes [26].

## 4. Discussion

This umbrella review yields two main findings: first, the modest but significant effectiveness of mobile app and long-term eHealth interventions, and second, the inconclusive evidence for stand-alone web-based approaches.

Specifically, mobile app interventions, particularly those delivered via smartphones, significantly reduce body weight compared to usual care or minimal interventions (MD = −1.32 kg; 95% CI: −2.54 to −0.11). Additionally, long-term eHealth interventions (≥12 months) achieved statistically significant weight reduction (MD = −1.13 kg; 95% CI: −2.17 to −0.09). Similarly, eHealth interventions resulted in significant reductions in BMI (MD = −0.58 kg/m^2^) and waist circumference (MD = −1.53 cm). However, web-based interventions, regardless of human support, did not yield statistically significant weight loss compared to controls. Despite the favorable direction of effect for web-based interventions with human support, their confidence intervals crossed the null value. Moderate to high heterogeneity (I^2^ > 80%) was evident across most analyses, with notably high heterogeneity observed in web-based interventions without human support.

### 4.1. Comparison with Previous Umbrella Reviews

These findings refine and update the results reported by Sorgente et al. (2017) [29], who found that web-based interventions yielded small but statistically significant weight reductions in overweight and obese individuals, particularly when personalized feedback was included. Similarly, Singh et al. (2024) [30] reported an average weight reduction of −1.89 kg (95% CI: −2.42 to −1.36) across diverse digital health strategies, alongside increased physical activity (+1329 steps/day) and reduced sedentary behavior (−426 min/week), reinforcing the overall effectiveness of digital interventions.

While our results align directionally with these findings, our review provides a more precise and specific conclusion: mobile apps are the most consistent modality for effective weight loss, considering that recent data show that web-based interventions, even with added human support, generally failed to achieve statistical significance in pooled analyses for weight loss.

### 4.2. Context and Need for Research in Diverse Settings

As also highlighted by König et al. (2025) [31], most studies included in both our review and prior literature were conducted in high-income countries. This imbalance creates a notable gap in evidence from lower- and middle-income regions. This evidence gap is especially acute in Latin America and the African continent, regions that were significantly underrepresented in the included studies.

To enhance the policy relevance and generalizability of eHealth research, merely conducting more studies in these underrepresented regions is not sufficient; the interventions themselves must be contextually appropriate. Future research should, therefore, prioritize the development and validation of culturally adapted interventions, ensuring that content, language, and behavioral cues are resonant with local social norms and dietary habits. Furthermore, addressing the digital divide is critical. Study protocols must incorporate digital literacy strategies, such as initial user training and ongoing technical support, to ensure that technological barriers do not prevent access for the most vulnerable populations [32,33,34]. Only by addressing both cultural context and digital literacy can the full potential of these technologies be realized equitably on a global scale.

Future studies must also address vulnerable populations and individuals managed within PHC, which present specific needs and challenges for intervention delivery and adherence. Such studies should evaluate not only efficacy but also barriers to access, digital literacy, cultural adaptation, and integration with existing public health strategies.

### 4.3. Factors Influencing Digital Intervention Outcomes

In addition to quantitative results, qualitative insights from included reviews help explain differences in adherence and sustainability of digital interventions. Mobile health interventions focusing on lifestyle self-monitoring commonly reported improved user autonomy, empowerment, and engagement due to easy accessibility and reduced dependence on in-person contacts [18]. However, maintaining long-term engagement appeared challenging without sufficient human interaction, especially in comprehensive interventions targeting multiple behaviors such as nutrition, physical activity, and mental health [19,20]. Similarly, web-based programs highlighted the critical role of structured behavioral elements (goal-setting, self-monitoring, and particularly regular personalized feedback from health professionals) as key determinants of effectiveness [9,21,22,24,25,26].

These qualitative insights underline the importance of strategically designed interventions that sustain engagement through ongoing human support and user-centered features.

### 4.4. Implications for Clinical Practice and Public Health Policy

The observed efficacy of mHealth interventions, particularly those incorporating behavior change components and long-term monitoring, underscores their potential as tools to support weight management within both primary care and broader public health strategies.

However, the long-term sustainability and cost-effectiveness of these interventions are critical for their integration into health systems. While the literature demonstrates effectiveness, most interventions are evaluated within time-limited, grant-funded research contexts, leaving their long-term viability uncertain. Similarly, a significant gap exists in formal cost-effectiveness analyses. For policymakers and healthcare providers to make informed decisions, future research must move beyond efficacy and incorporate rigorous analyses of implementation models and cost-effectiveness.

The successful integration of these digital tools into public health systems requires more than just technological innovation. As noted by Cao et al. (2022) [32], digital health solutions must be embedded within broader health strategies, supported by robust governance, ongoing workforce training, investment in digital infrastructure, and systematic evaluation. Examining emerging public policy models illustrates these principles in action.

For instance, one direct public eHealth solution is the “Peso Saudável” (Healthy Weight) functionality, integrated into the Brazilian Health System’s eHealth platform (Conecte SUS). This resource provides users with a tool for self-monitoring their weight and guidance on making healthy lifestyle changes, representing an innovative strategy to support obesity care within the primary healthcare system [35].

In England, the National Health Service (NHS) Digital Weight Management Programme provides a contrasting commissioning model. This 12-week program, accessible via primary care referral, delivers both behavioral and nutritional support. An evaluation of over 63,000 referrals demonstrated clinically meaningful weight loss (an average of −2.2 kg for program finishers and −3.9 kg for completers), with equitable effectiveness observed across diverse socioeconomic and ethnic groups [36].

Despite these advances, challenges persist. In low- and middle-income settings, such as with the NUTRES SMS program in Mexico, implementation was challenged by barriers, including limited mobile access and poor connectivity, underscoring how the digital divide can limit the real-world impact of even well-received interventions [37]. Furthermore, while large-scale deployments, such as “Peso Saudável,” are valuable case studies, many still lack published, peer-reviewed efficacy data.

In this context, pilot programs, feasibility studies, and contextual adaptations are essential to ensure that digital interventions are equitable, accessible, and effective across diverse populations. The success of public and community mHealth interventions depends not only on the technology but on its deep integration with human support, its adaptation to local infrastructure, and a steadfast commitment to evaluation and equity to maximize its population-level impact.

### 4.5. Equity and Accessibility Considerations

One limitation of digital interventions, as reported by Lozada-Tequeanes et al. (2024) [37], is the risk of excluding individuals with low digital literacy, limited access to devices, or inaccessible connectivity. In this context, researchers, developers, and policymakers must prioritize cultural tailoring, simplified interfaces, inclusive design, and public policies of digital inclusion to avoid deepening health disparities.

As reported by Mahou et al. (2021) [33], individuals with higher income and education levels were significantly more likely to use digital health services, underscoring socio-economic inequalities in access. To truly promote digital health equity, as emphasized by Lawrence (2022) [34], it is essential to address barriers at multiple levels. At the design stage, technologies must consider the lived experiences and preferences of diverse populations, avoiding algorithmic or interface-level biases that may marginalize specific groups. At the implementation level, interventions must be aligned with the realities of healthcare infrastructure and integrated into trusted points of care. Engagement strategies should also acknowledge user concerns, such as privacy, autonomy, and digital fatigue, especially among historically underserved populations. Given the complexity of this reality, equity-focused research and inclusive design are thus not only ethical imperatives but also efficacy determinants.

### 4.6. The Emerging Role of Artificial Intelligence in Digital Weight Management

Although artificial intelligence (AI) was not a focus of the interventions included in this umbrella review, recent developments in AI-driven health technologies have begun to reshape the landscape of digital weight management. Tools leveraging AI, such as adaptive feedback systems, chatbots, and predictive analytics, promise greater personalization and real-time adjustment to user needs, potentially improving adherence and outcomes compared to conventional digital strategies. In addition to these software-based solutions, recent advances also encompass hardware platforms, such as smartwatches and other wearable devices, which can provide real-time monitoring and adaptive feedback when integrated with AI-driven digital health interventions [38,39,40].

Despite this potential, the current evidence base for AI-enabled interventions in weight management remains limited, with few robust randomized controlled trials or high-quality systematic reviews available for critical appraisal. This fact is likely due to the novelty of these technologies and the time required for rigorous evaluation and reporting in the literature [41].

This umbrella review highlights significant limitations within the current eHealth literature, such as inconsistent evidence for web-based interventions and high methodological heterogeneity across most analyses. As the field advances toward integrating more advanced technologies, such as AI, it is imperative that these existing shortcomings are not replicated. Evaluating AI presents its own critical challenges, particularly regarding transparency, reproducibility, and algorithmic bias [41]. To pre-emptively address these issues, future research must adopt rigorous methodologies [12]. For instance, to improve reproducibility and mitigate bias, study protocols should include a systematic and justified selection of the AI models analyzed, prioritize experiments using direct Application Programming Interfaces over public web interfaces, and standardize deterministic model settings (e.g., ‘temperature’ = 0). By implementing such standards from the outset, the field has an opportunity to build a trustworthy and equitable evidence base for AI in weight management, preventing the cycle of inconclusive findings that have affected other areas of eHealth.

### 4.7. Methodological Considerations and Limitations

One important methodological consideration in this umbrella review concerns the inclusion of pharmacological interventions as part of the conventional treatment in comparator groups. Because information regarding the use of pharmacotherapy in comparators tends to be underreported or not explicitly detailed in systematic reviews, we opted a priori not to exclude reviews solely based on insufficient specification about medication use. While our critical appraisal indicated a low risk of bias from this source across the included evidence, the lack of detailed reporting may limit the ability to exclude potential confounding from unreported pharmacotherapy entirely. This approach was chosen to ensure comprehensiveness and external validity. However, future reviews should encourage the systematic reporting of all active components in comparator arms.

Furthermore, this review used comprehensive search strategies, dual independent screening, and overlap quantification. Subgroup and moderator analyses helped clarify effect sizes and their potential modifiers. Still, the high heterogeneity, reliance on studies from high-income settings, and variability in intervention designs remain significant limitations. Most included studies had short-term interventions, and only a minority evaluated long-term outcomes. The sustainability of weight loss beyond the intervention period remains uncertain.

## 5. Conclusions

This umbrella review confirms that mobile app interventions and long-term eHealth strategies produce modest but significant reductions in body weight, BMI, and waist circumference in overweight and obese adults, whereas the evidence for stand-alone web-based interventions remains inconclusive. The current evidence base is largely derived from studies in high-income countries, with limited representation from low- and middle-income regions and primary care settings.

We therefore issue a clear call to action for researchers, practitioners, and policymakers. Researchers must prioritize culturally tailored, methodologically rigorous studies in underrepresented and vulnerable populations, incorporating digital literacy strategies and robust assessments of sustainability and cost-effectiveness. Emerging technologies, such as AI models in the context of digital health integrations, highlight the need for high-quality trials and comprehensive reviews to clarify the impact, safety, and equity of AI-enabled interventions. Practitioners should integrate evidence-based digital tools as adjuncts to comprehensive weight management strategies, ensuring equitable access and sustained patient engagement. Policymakers have a responsibility to invest in the development, evaluation, and sustainable implementation of digital health solutions, establishing supportive frameworks for funding, data governance, and digital infrastructure.

Ultimately, collaborative efforts among researchers, practitioners, and policymakers remain essential to fully realize the population-level benefits of digital interventions in addressing the global challenge of obesity.

## Figures and Tables

**Table 1:** Characteristics of selected reviews.

Reference	N Studies	Countries/Regions of Studies (N)	Pub. Years	Population (C, I)	Age (Years)	Sex (M:F)	Intervention(s)	Comparison(s)	Outcome(s)	Main Effect Size
[18]	20	US (12), UK (2), AU (3), NZ (1), SK (1), FI (1)	2007–2019	2196 (1064, 1132)	20.5–59.8 (range)	NR	eHealth interventions (Smartphone, PDA, Web)	Conventional care Paper records Wait-list	Body weight reduction (kg) Intervention adherence	Weight: MD = −1.78 (−2.70 to −0.85) Adherence: RR = 0.78 (0.63 to 0.96)
[10]	20	US (15), UK (2), AU (2), CN (1)	2011–2016	2318 (1162, 1156)	18–70 (range)	NR	Mobile health (mHealth) interventions (SMS and/or Mobile Apps)	Conventional care No intervention	Body weight reduction (kg) BMI (kg/m^2^)	Weight: WMD = −2.35 (−2.84 to −1.87) BMI: WMD = −0.77 (−1.01 to −0.52)
[19]	34	US (12), AU (6), NZ (2), FI (2), JP (2), SG (2), TH (1), IR (1), DO (1), IS (1), NL (1), KR (1), SE (1), RO (1)	2013–2023	5379 (2161, 3403)	39 (mean; SD 12.5)	1360:2340 *	eHealth interventions (Mobile Apps, SMS, Web, Wearables)	Wait-list Standard care Sham control No intervention	Body weight reduction (kg) Perceived stress reduction Diet quality Self-reported physical activity	Weight: MD = −1.70 (−2.45 to −0.95) Stress: SMD = −0.32 (−0.52 to −0.12)
[20]	6	US (4), FI (1), UK (1)	2008–2013	632 (312, 320)	38–53 (range of means)	73:559	Mobile health (mHealth) interventions (PDA, SMS, Mobile Apps)	Paper records Printed materials No intervention	Body weight reduction (kg) Waist circumference (cm)	Weight (PDA): WMD = −1.09 (−2.12 to −0.05) Weight (mHealth): WMD = −1.78 (−2.92 to −0.63)
[21]	15	AU (8), US (7)	2001–2017	2630 (882, 1748)	44.8 (mean; SD 0.9)	1173:1253	Web-based behavioral interventions	Face-to-face modalities Self-help, Wait-list	Body weight reduction (kg) Weight maintenance (follow-up) Dropout rates	Weight (vs WL): MD = −4.32 (−5.08 to −3.57) Weight (vs Self-help): MD = −4.31 (−5.22 to −3.41)
[9]	18	US (17), UK (1)	2001–2008	5700 (2570; 3130)	32–51 (range of means)	NR †	Web-based behavioral interventions	Minimal intervention Wait-list Printed materials Face-to-face programs	Body weight reduction (kg) Weight maintenance (kg)	Weight (vs Min. Interv.): SMD = 0.73 (not sig.) Weight (vs Edu-only): WMD = 2.24 (1.27 to 3.21)
[22]	84	North America (63), Europe/UK (10), Oceania (8), Asia (2), Multisite (1)	2001–2014	24,010 (3707, 3906)	35–65 (range of means)	NR ‡	eHealth interventions (Web, Email, Mobile Apps, SMS)	Conventional care Minimal intervention No intervention Other eHealth Face-to-face modalities	Body weight reduction BMI Body fat (%) Waist circumference (cm)	Weight (vs NI): MD = −2.70 (−3.33 to −2.08) Weight (vs Min. Interv.): MD = −1.40 (−1.98 to −0.82)
[23]	18	US (6), UK (3), ES (3), JP (2), BE (1), DE (1), NZ (1), CH (1)	2013–2023	2703 (NR)	18–60 (range of means) §	NR	Mobile health (mHealth) interventions (Mobile Apps)	Conventional care Printed materials Wait-list Minimal intervention	Body weight reduction (kg) BMI (kg/m^2^) Waist circumference (cm)	Weight: MD = −1.40 (−2.13 to −0.67) BMI: MD = −0.39 (−0.63 to −0.15) WC: MD = −1.32 (−2.21 to −0.43)
[24]	7	US (4), AU (1), DE (1), SK (1)	2009–2014	779 (371, 408)	41.4–57.6 (range of means)	NR	Web-based behavioral interventions	Conventional care No intervention	Body weight reduction (kg)	Weight: WMD = 0.56 (−3.47 to 4.59) (not sig.)
[25]	28	US (7), SA (4), UK (4), IN (3), CN (3), SG (2), JP (2), KR (1), CA (1), TH (1), BD (1), AU (1), PK (1), LK (1)	1999–2024	14,398 (NR)	39–66 (range of means)	NR	eHealth interventions (Mobile Apps, SMS, Web)	Conventional care Printed materials	Body weight reduction (kg) BMI (kg/m^2^) Waist circumference (cm) HbA1c (%) Other metabolic outcomes	Weight: MD = −1.74 (−2.37 to −1.11) BMI: MD = −0.58 (−0.83 to −0.32) WC: MD = −1.58 (−2.11 to −1.04)
[26]	11	NR	2007–2018	1662 (821, 841)	18–65 (range)	NR	Web-based behavioral interventions	Face-to-face interventions Wait-list control	Body weight reduction (kg) BMI (kg/m^2^) Waist circumference (cm) Body fat (%) Diet quality Physical activity	Weight (<6 m): MD = −2.13 (−2.71 to −1.55) Weight (Overall): MD = −0.77 (not sig.)

AU, Australia; BD, Bangladesh; BE, Belgium; BMI, Body Mass Index; C, Control Group; CA, Canada; CC, Conventional Care; CH, Switzerland; CI, Confidence Interval; CN, China; DE, Germany; DO, Dominican Republic; ES, Spain; FI, Finland; HbA1c, Hemoglobin A1c; I, Intervention Group; I^2^, I-squared heterogeneity statistic; IN, India; IR, Iran; IS, Iceland; JP, Japan; KR, South Korea; LK, Sri Lanka; m, months; MD, Mean Difference; Min. Interv., Minimal Intervention; N, Number; NI, No Intervention; NL, Netherlands; NR, Not Reported; NZ, New Zealand; PA, Physical Activity; PDA, Personal Digital Assistant; PK, Pakistan; PM, Printed Materials; PR, Paper Records; RO, Romania; RR, Risk Ratio; SA, Saudi Arabia; SC, Standard Care; SD, Standard Deviation; SE, Sweden; SG, Singapore; not sig., not significant. SMS, Short Message Service; TH, Thailand; UK, United Kingdom; US, United States; vs., versus; WC, Waist Circumference; WL, Wait-list; WMD, Weighted Mean Difference. * Absolute numbers of males and females among the 27 studies that reported this information. † Sex distribution not reported in aggregate; the article states that at least 77% of participants were female. Absolute numbers were not provided. ‡ Range of mean ages among the 69 studies that reported mean age. § Mean female proportion across included studies: 74.8%. Absolute numbers were not provided. None of the comparator groups were described as receiving systematic pharmacological interventions. All comparators involved behavioral, educational, usual care, minimal intervention, or wait-list controls, as detailed in the table.

**Table 2:** Representative effect sizes from individual studies.

Reference	Population	Intervention	Comparison Group	Outcome	Effect Size	95% CI	*p* Value	I^2^ (%)	Publication Bias	Strength of Evidence	GRADE
[18]	Adults with overweight/obesity	mHealth lifestyle self-monitoring	Usual care, paper record, wait-list	Weight loss (kg)	−0.37 (Cohen’s d); −1.78 kg	−0.54 to −0.19 (d); −2.70 to −0.85 (kg)	<0.001	84.6	Funnel plot and Egger’s test: bias detected (*p* = 0.016)	Moderate	NR
	Adults with overweight/obesity	mHealth lifestyle self-monitoring	Usual care, paper record, wait-list	Adherence (risk of dropout)	RR = 0.78	0.63 to 0.96	0.049	37.8	Funnel plot and Egger’s test: bias detected (*p* = 0.008)	Moderate	NR
[10]	Adults with overweight/obesity	Mobile health (mHealth) interventions	Usual care/education	Weight loss (kg)	−2.35 kg	−2.84 to −1.87	<0.001	94	Mild bias; funnel plot, no formal test	NR	NR
	Adults with overweight/obesity	Mobile health (mHealth) interventions	Usual care/education	BMI change (kg/m^2^)	−0.77 kg/m^2^	−1.01 to −0.52	<0.001	95	NR	NR	NR
[19]	Adults at risk of NCDs, including overweight/obese	Holistic mHealth interventions	Usual care, education, wait-list, or sham control	Weight change (kg)	−1.70 kg	−2.45 to −0.95	<0.001	89	Egger’s test: no bias (*p* = 0.95)	Small to moderate (main text)	NR
	Adults at risk of NCDs, including overweight/obese	Holistic mHealth interventions	Usual care, education, wait-list, or sham control	Perceived stress (SMD)	−0.32 (SMD, Hedges’ g)	−0.52 to −0.12	0.49	14.5	Not assessed (not enough studies)	Small (main text)	NR
	Adults at risk of NCDs, including overweight/obese	Holistic mHealth interventions	Usual care, education, wait-list, or sham control	Diet quality (SMD)	0.21 (SMD, Hedges’ g)	−0.15 to 0.56	0.25	62.3	Not assessed (not enough studies)	Not significant	NR
	Adults at risk of NCDs, including overweight/obese	Holistic mHealth interventions	Usual care, education, wait-list, or sham control	MVPA (SMD)	0.21 (SMD, Hedges’ g)	−0.25 to 0.67	0.37	74.3	Not assessed (not enough studies)	Not significant	NR
[20]	Adults with overweight/obesity	Mobile health (mHealth) interventions	Usual care or paper-based diary	Weight loss (kg)	−1.09 kg (WMD)	−2.12 to −0.05	0.04	49.6	No bias: Begg’s test and funnel plot	NR	NR
[21]	Adults with overweight/obesity	Intensive Contact Web-based interventions	Wait-list	Weight loss (kg)	−1.86 (MD, kg)	−3.61 to −0.12	0.04	80.2	Funnel plot: possible bias, not formal	NR	NR
[9]	Adults with overweight/obesity	Web-based interventions	Control/minimal intervention	Weight loss (kg)	−0.73 (MD, kg)	−0.06 to 1.51	0.07	84.4	Funnel plot: possible bias, not formal test	NR	NR
[22]	Adults with overweight/obesity	eHealth interventions (web, mobile, etc.)	No intervention control	Weight loss (kg)	−2.70 (MD, kg)	−3.33 to −2.08	<0.00001	49	Funnel plot: no bias detected	NR	NR
	Adults with overweight/obesity	eHealth interventions (web, mobile, etc.)	Minimal intervention (e.g., written self-help)	Weight loss (kg)	−1.40 (MD, kg)	−1.98 to −0.82	<0.0001	72	Funnel plot: no bias detected	NR	NR
	Adults with overweight/obesity	eHealth with extra features/technologies	Standard eHealth interventions	Weight loss (kg)	1.46 (MD, kg)	0.80 to 2.13	<0.001	60	Funnel plot: no bias detected	NR	NR
	Adults with overweight/obesity	eHealth (mainly web/email)	Control/minimal intervention	Weight change (kg)	−0.27 (MD, kg)	−0.96 to 0.42	0.44	0	NR	NR	NR
[23]	General population with overweight/obesity	Mobile app-based mHealth	Minimal intervention, no intervention, or usual care	Weight change (kg)	−2.10 (MD, kg)	−2.79 to −1.41	<0.00001	75	NR	Moderate	Moderate
[24]	Adults with overweight/obesity	Web-based digital health interventions	Non-web-based control	Weight loss (kg)	0.56 (WMD, kg)	−3.47 to 4.59	0.789	74.8	No bias: Begg’s *p* = 0.67; Egger’s *p* = 0.78	NR	NR
[25]	Adults with prediabetes (majority overweight/obese)	Digital health lifestyle interventions	Non-digital control	Weight change (kg)	−1.74 (MD, kg)	−2.37 to −1.11	<0.01	84.4	Funnel plot and Egger’s test: no bias (*p* = 0.23)	Moderate	Moderate
	Adults with prediabetes (majority overweight/obese)	Digital health lifestyle interventions	Non-digital control	BMI (kg/m^2^)	−0.58 (MD)	−0.83 to −0.32	<0.01	78	NR	Low	Low
	Adults with prediabetes (majority overweight/obese)	Digital health lifestyle interventions	Non-digital control	Waist circumference (cm)	−1.58 (MD)	−2.11 to −1.04	<0.01	62	NR	Low	Low
	Adults with prediabetes (majority overweight/obese)	Digital health lifestyle interventions	Non-digital control	HbA1c (%)	−0.07 (MD)	−0.09 to −0.05	<0.01	0	NR	Moderate	Moderate
	Adults with prediabetes (majority overweight/obese)	Digital health lifestyle interventions	Non-digital control	More than 5% weight loss (log odds)	1.49 (log odds)	1.20 to 1.78	<0.01	NR	NR	Moderate	Moderate
[26]	Adults with overweight/obesity	Web-based digital health interventions	Offline or face-to-face interventions, wait list	Weight change (kg)	−0.77 (MD, kg)	−2.16 to 0.62	0.28	94	Not a major issue (GRADE, not formal test)	Moderate	Moderate
	Adults with overweight/obesity	Web-based digital health interventions	Offline or face-to-face interventions, wait list	BMI change (kg/m^2^)	−0.12 (MD)	−0.64 to 0.41	0.65	NR	Not a major issue (GRADE, not formal test)	Moderate	Moderate
	Adults with overweight/obesity	Web-based digital health interventions	Offline or face-to-face interventions, wait list	Waist circumference (cm)	−0.54 (MD, cm)	−5.17 to 4.10	NR	NR	NR	NR	NR
	Adults with overweight/obesity	Web-based digital health interventions	Offline or face-to-face interventions, wait list	Body fat (%)	−1.40 (%)	−2.93 to 0.13	NR	NR	NR	NR	NR

CC, Conventional/Usual Care; CI, Confidence Interval; Cohen’s d, standardized mean difference (Cohen’s method); Edu, Education; GRADE, Grading of Recommendations, Assessment, Development and Evaluations; HbA1c, Hemoglobin A1c; Hedges’ g, standardized mean difference (Hedges’ method); I^2^, I-squared heterogeneity statistic; log odds, logarithm of odds ratio; MD, Mean Difference; mHealth, Mobile Health; Min. Interv., Minimal Intervention; MVPA, Moderate-to-Vigorous Physical Activity; NCD, Non-communicable Disease; NI, No Intervention; NR, Not Reported; PM, Paper-based Materials (records/diary); RR, Risk Ratio; SMD, Standardized Mean Difference; vs., versus; WC, Waist Circumference; WL, Wait-list; WMD, Weighted Mean Difference; BMI, Body Mass Index. Note: The gray shading is used to improve readability only. It does not represent any analytical meaning.

**Table 3:** Moderator analyses.

Meta-Analysis (Year)	Moderators Tested	Main Findings/Results	Significant Moderators
[18]	Type of intervention (mHealth: smartphone, PDA, web-based) Type of control group (usual care, paper record, wait-list) Duration of intervention (≤3, 6, ≥12 months) Participant characteristics (age, baseline weight, BMI, waist circumference)	mHealth self-monitoring interventions produced moderate weight loss vs. controls (SMD = −0.37; 95% CI: −0.54 to −0.19; I^2^ = 84.6%). Greatest effect for smartphone interventions and when usual care was control. Short-term interventions (≤3 m) more effective. Higher adherence vs. paper records. No significant moderation by age, baseline weight, or BMI. Publication bias present.	Intervention type (smartphone) Comparator (usual care) Intervention duration (≤3 months)
[10]	Duration of intervention (3–4, 6, 9, ≥12 months) Participant characteristics (mean age, baseline BMI, type of obesity) Study factor (year of publication)	mHealth interventions in adults resulted in significant weight loss vs. controls (WMD = −2.35 kg; 95% CI: −2.84 to −1.87; I^2^ = 94%). Effect greater at 6–9 months, attenuated at ≥12 m. BMI also reduced. No significant subgroup moderators. Short-term effect modest; mild publication bias.	Intervention duration
[19]	Type of control group (wait-list/standard care vs. sham) Target population (general adults vs. overweight/obese) Risk of bias Duration of intervention Delivery mode Human support Outcome measurement (device-based vs. self-report for physical activity)	Holistic mHealth interventions (PA, diet, mental health) yielded significant weight loss (MD = −1.70 kg) and reduced stress vs. controls. No significant effect on physical activity or diet quality. Greater effect vs. wait-list/standard care. Most studies short-term; some/high risk of bias.	Control group type (for weight loss) Target population (for diet quality)
[20]	Type of intervention (device type: mobile phone, PDA) Duration of intervention (≤6 months, >6 months)	Mobile device interventions (phones, PDAs, apps) produced modest weight loss vs. controls (WMD = −1.09 kg). Greater loss with mobile phone interventions. No difference by duration. No publication bias.	Device type (mobile phone)
[21]	Type of intervention (IC-W, MC-W, GSH-W, SH-W, SH, wait-list) Feedback features (frequency/personalization, provider: human/machine) Duration of intervention Participant characteristics (age, baseline weight, sample size) Study quality (risk of bias)	Intensive Contact Web-based (IC-W) programs with frequent professional feedback were most effective (MD = −1.86 kg vs. wait-list). All web-based interventions outperformed wait-list. Only intervention length was a significant moderator. Long-term data limited; dropout rates similar.	Feedback frequency/personalization Intervention length
[9]	Type of intervention (web-based with behavioral features/feedback, education-only, control/minimal, face-to-face) Adherence/usage (log-ins, self-monitoring, chat/board attendance) Outcome focus (weight loss vs. maintenance)	Web-based interventions led to modest weight loss vs. minimal/control (SMD = 0.73; 95% CI: −0.06 to 1.51). Enhanced behavioral features increased efficacy (WMD = 2.24 kg). Similar maintenance to face-to-face. High attrition and short follow-up.	Behavioral features Feedback Adherence/usage
[22]	Type of intervention (web, mobile, computer, with/without non-eHealth components) Type of control group (control, minimal, standard care, face-to-face) Technological features (additional features/technologies, monitoring devices) Duration of intervention Study quality (ITT analysis, retention) Participant characteristics (publication year, continent, gender, baseline BMI)	eHealth interventions resulted in modest weight loss vs. controls (MD = −2.70 kg), stronger with additional non-eHealth features. Extra features (self-monitoring, feedback) increased effect. No significant effect on weight maintenance. No publication bias.	Intervention type Additional features Monitoring devices Intervention duration Study quality Intent-to-treat (ITT) analysis Retention Publication year Continent
[23]	Duration of follow-up (medium, long term) Population (adolescents, adults) Type of control group (no/minimal intervention) Outcomes measured (physical activity, anthropometric measures, quality of life, self-efficacy, well-being, dietary behavior, adverse events)	mHealth smartphone interventions in adults produced significant weight loss at medium- and long-term follow-up (WMD = −1.79 kg and −1.35 kg). Secondary improvements in physical activity, diet, and quality of life were mixed. Moderate-high heterogeneity; safe profile.	No significant moderators identified Subgroup and meta-regression analyses did not reveal significant effect modification by follow-up duration, population, type of control group, or outcome measured.
[24]	Participant characteristics (age, country: USA vs. other) Duration of intervention (12, 16, 24 weeks) Study quality/methodology (intention-to-treat analysis, Jadad score, drop-out rate) Intervention features (interactive web-based, feedback included, goal-setting included)	Web-based interventions showed no significant effect on weight loss vs. controls (WMD = 0.56 kg). Feedback and goal-setting features improved outcomes. Certainty limited by high heterogeneity and poor methodological quality.	Age group Intent-to-treat (ITT) analysis Feedback Goal-setting Jadad score Country (USA vs. others)
[25]	Continent (North America, Asia, Europe) Duration of intervention Contact type (face-to-face, stand-alone, remote, combined) Frequency of digital contact Participant characteristics (baseline BMI) Technological features (digital scale, self-monitoring of weight, provider type, tracking technologies) Type of control group (usual care, enhanced care, active)	Digital health lifestyle interventions (DHLI) for prediabetes yielded significant weight loss vs. non-digital (MD = −1.74 kg). Greater effect with face-to-face elements, higher BMI, and self-monitoring. Secondary benefits for BMI, waist, HbA1c. Most studies at risk of bias.	Continent Study duration Contact type Frequency of digital contact Baseline BMI Digital scale Self-monitoring of weight
[26]	Type of control group (active intervention vs. wait-list) Duration of follow-up (<6 vs. ≥6 months)	Web-based interventions had no significant difference vs. offline in weight loss (MD = −0.77 kg). Greater short-term (<6 m) effect. No long-term benefit or difference in other outcomes. Moderate certainty; high attrition.	Control group type Follow-up duration

CI, Confidence Interval; DHLI, Digital Health Lifestyle Intervention; eHealth, Electronic Health; GSH-W, Guided Self-Help Web-based; HbA1c, Hemoglobin A1c; I^2^, I-squared heterogeneity statistic; IC-W, Intensive Contact Web-based; ITT, Intent-to-Treat; m, months; MC-W, Moderate Contact Web-based; MD, Mean Difference; mHealth, Mobile Health; NCD, Non-communicable Disease; NR, Not Reported; PA, Physical Activity; PDA, Personal Digital Assistant; RR, Risk Ratio; SH, Self-Help; SH-W, Self-Help Web-based; SMD, Standardized Mean Difference; vs., versus; WL, Wait-list; WMD, Weighted Mean Difference; BMI, Body Mass Index.

## Data Availability

The data presented in this study are derived from previously published systematic reviews and meta-analyses available in public domain databases (Cochrane Library, LILACS, PubMed, ScienceDirect, and Web of Science). The detailed electronic search strategies are provided in File S1. The original contributions and extracted data are included in the article and Supplementary Material. Additional data or materials can be requested from the corresponding author.

## Supplementary Materials

The following supporting information can be downloaded at https://www.mdpi.com/article/10.3390/ijerph22071152/s1, Table S1: PRISMA Checklist; File S1: Electronic Search Strategy; File S2: R Scripts for Cohen’s Kappa Coefficient Calculation; Table S2: Reasons for Full-Text Exclusion; Table S3: Overlap of primary studies; File S3: R Scripts for Data Extraction, Subgroup Selection, and Meta-Analyses.

## Abbreviations

The following abbreviations are used in this manuscript:

AI	Artificial Intelligence
AMSTAR 2	A MeaSurement Tool to Assess Systematic Reviews (version 2)
BIB	BibTeX file format
BMI	Body Mass Index
CCA	Corrected Covered Area
CI	Confidence Interval
COVID-19	Coronavirus Disease 2019
DALYs	Disability-Adjusted Life Years
eHealth	Electronic Health
GDP	Gross Domestic Product
GRADE	Grading of Recommendations, Assessment, Development and Evaluation
IMB	Information-Motivation-Behavioral Skills
ITT	Intention-To-Treat
JBI-MAStARI	Joanna Briggs Institute Meta-Analysis of Statistics Assessment and Review Instrument
MD	Mean Difference
mHealth	Mobile Health
NBIB	Native BibTeX format
PDA	Personal Digital Assistant
PHC	Primary Healthcare
PICOS	Population, Intervention, Comparator, Outcome, Study design
PRISMA	Preferred Reporting Items for Systematic Reviews and Meta-Analyses
PROSPERO	International Prospective Register of Systematic Reviews
R	Programming language and statistical computing environment
RoB	Risk of Bias
ROBINS-I	Risk Of Bias In Non-Randomized Studies-of Interventions
RR	Risk Ratio
SMD	Standardized Mean Difference
SUS	Sistema Único de Saúde
