# Supplementary material for: Mobile and Web Apps for Weight Management in Overweight and Obese Adults: An Updated Umbrella Review and Meta-Analysis
Source: Int J Environ Res Public Health. 2025 Jul 21;22(7):1152. doi: 10.3390/ijerph22071152 (PMC12294216; doi:10.3390/ijerph22071152)
Supplement: Supplementary file 1 [file ijerph-22-01152-s001.zip › File S1. Electronic Search Strategy.docx.pdf]

File S1. Electronic Search Strategy

Cochrane, february 2nd, 2025:

(obes* OR “overweight” OR “body NEXT weight”)
AND
(mobile NEXT application* OR web-based NEXT intervention* OR "digital NEXT intervention" OR “digital NEXT health” OR “mHealth” OR “telehealth”)
AND
(“weight NEXT loss” OR “body mass index” OR “BMI” OR “waist NEXT circumference” OR “body NEXT fat”)

LILACS, february 2nd, 2025:

(ti:
("obes*" OR "overweight" OR "body weight")
AND ti:
("mobile application*" OR "web-based intervention*" OR "mHealth" OR "digital intervention*" OR "digital health" OR "telehealth")
AND ti:
("weight loss" OR “body mass index” OR "BMI" OR "waist circumference" OR "body fat")
AND ti:
("systematic review" OR "meta-analysis"))
OR

(ab:
("obes*" OR "overweight" OR "body weight")
AND ab:
("mobile application*" OR "web-based intervention*" OR "mHealth" OR "digital intervention*" OR "digital health" OR "telehealth")
AND ab:
("weight loss" OR “body mass index” OR "BMI" OR "waist circumference" OR "body fat")
AND ab:
("systematic review" OR "meta-analysis"))

PubMed, february 2nd, 2025:

(("obes*"[Title/Abstract] OR "overweight"[Title/Abstract] OR "body weight"[Title/Abstract])
AND
("mobile application*"[Title/Abstract] OR "web-based intervention*"[Title/Abstract] OR "mHealth"[Title/Abstract] OR "digital intervention*"[Title/Abstract] OR "telehealth"[Title/Abstract])
AND

("weight loss"[Title/Abstract] OR "Body Mass Index" [Title/Abstract] OR "BMI" [Title/Abstract] OR "waist circumference"[Title/Abstract] OR "body fat"[Title/Abstract])
AND
("review"[Publication Type] OR "systematic review"[Publication Type] OR "meta-analysis"[Publication Type]))

Science Direct, february 2nd, 2025:

("obesity" OR "overweight")
AND
("mobile application" OR "web-based intervention" OR "digital health")
AND
("weight loss" OR "BMI" OR "waist circumference" OR "body fat")

Web of Science, february 2nd, 2025:

(TI=
("obes*" OR "overweight" OR "body weight")
AND TI=
("mobile application*" OR "web-based intervention*" OR "mHealth" OR "digital intervention*" OR "digital health" OR "telehealth")
AND TI=
("systematic review" OR "meta-analysis")
AND TI=
("weight loss" OR “body mass index” OR "BMI" OR "waist circumference" OR "body fat"))
OR

(AB=
("obes*" OR "overweight" OR "body weight")
AND AB=
("mobile application*" OR "web-based intervention*" OR "mHealth" OR "digital intervention*" OR "digital health" OR "telehealth")
AND AB=
("systematic review" OR "meta-analysis")
AND AB=
("weight loss" OR “body mass index” OR "BMI" OR "waist circumference" OR "body fat"))
