# Supplementary material for: Mobile and Web Apps for Weight Management in Overweight and Obese Adults: An Updated Umbrella Review and Meta-Analysis
Source: Int J Environ Res Public Health. 2025 Jul 21;22(7):1152. doi: 10.3390/ijerph22071152 (PMC12294216; doi:10.3390/ijerph22071152)
Supplement: Supplementary file 1 [file ijerph-22-01152-s001.zip › File S2. R Scripts for Cohen’s Kappa Coefficient Calculation.docx.pdf]

File S2. R Scripts for Cohen's Kappa Coefficient Calculation

Title and Abstract Screening

Load necessary package

```
install.packages("irr")
library(irr)
```

Create the classification matrix based on screening decisions

```
FFSC <- c("Excluded", "Excluded", "Excluded", "Excluded", "Excluded", "Excluded",
"Excluded", "Excluded", "Excluded", "Excluded", "Excluded", "Excluded", "Excluded",
"Excluded", "Excluded", "Excluded", "Excluded", "Excluded", "Excluded", "Excluded",
"Excluded", "Excluded", "Excluded", "Excluded", "Excluded", "Excluded", "Excluded",
"Excluded", "Excluded", "Excluded", "Excluded", "Excluded", "Excluded", "Excluded",
"Excluded", "Excluded", "Excluded", "Excluded", "Excluded", "Excluded", "Excluded",
"Excluded", "Excluded", "Excluded", "Excluded", "Excluded", "Excluded", "Excluded",
"Excluded", "Excluded", "Excluded", "Excluded", "Excluded", "Excluded", "Excluded",
"Excluded", "Excluded", "Excluded", "Excluded", "Excluded", "Excluded", "Excluded",
"Excluded", "Excluded", "Excluded", "Excluded", "Excluded", "Excluded", "Excluded",
"Excluded", "Excluded", "Excluded", "Excluded", "Excluded", "Excluded", "Excluded",
"Excluded", "Excluded", "Excluded", "Excluded", "Excluded", "Excluded", "Excluded",
"Excluded", "Excluded", "Excluded", "Excluded", "Excluded", "Excluded", "Excluded",
"Excluded", "Excluded", "Excluded", "Excluded", "Excluded", "Excluded", "Excluded",
"Excluded", "Excluded", "Excluded", "Excluded", "Excluded", "Excluded", "Excluded",
"Excluded", "Excluded", "Excluded", "Excluded", "Excluded", "Excluded", "Excluded",
"Excluded", "Excluded", "Excluded", "Excluded", "Excluded", "Excluded", "Excluded",
"Excluded", "Excluded", "Excluded", "Excluded", "Excluded", "Excluded", "Excluded",
"Excluded", "Excluded", "Excluded", "Excluded", "Excluded", "Excluded", "Excluded",
"Excluded", "Excluded", "Excluded", "Excluded", "Excluded", "Excluded", "Excluded",
"Excluded", "Excluded", "Excluded", "Excluded", "Excluded", "Excluded", "Excluded",
"Excluded", "Excluded", "Excluded", "Excluded", "Excluded", "Excluded", "Excluded",
"Excluded", "Excluded", "Excluded", "Excluded", "Excluded", "Excluded", "Excluded",
"Excluded", "Excluded", "Excluded", "Excluded", "Excluded", "Excluded", "Excluded",
"Excluded", "Excluded", "Included", "Included", "Included", "Included", "Included",
"Included", "Included", "Included", "Included", "Included", "Included", "Included",
"Included", "Included", "Included", "Included", "Included", "Included", "Included",
"Included", "Included", "Included", "Included", "Included", "Included", "Included",
"Included", "Included", "Maybe", "Maybe", "Maybe", "Maybe", "Maybe", "Maybe",
"Maybe", "Maybe", "Maybe", "Maybe", "Excluded", "Excluded", "Maybe", "Excluded",
"Maybe", "Maybe", "Included", "Excluded", "Excluded", "Included", "Excluded",
"Excluded", "Excluded", "Maybe", "Excluded", "Maybe")
CPBA <- c("Excluded", "Excluded", "Excluded", "Excluded", "Excluded", "Excluded",
"Excluded", "Excluded", "Excluded", "Excluded", "Excluded", "Excluded", "Excluded",
```

*"Excluded", "Excluded", "Excluded", "Excluded", "Excluded", "Excluded", "Excluded",
"Excluded", "Excluded", "Excluded", "Excluded", "Excluded", "Excluded", "Excluded",
"Excluded", "Excluded", "Excluded", "Excluded", "Excluded", "Excluded", "Excluded",
"Excluded", "Excluded", "Excluded", "Excluded", "Excluded", "Excluded", "Excluded",
"Excluded", "Excluded", "Excluded", "Excluded", "Excluded", "Excluded", "Excluded",
"Excluded", "Excluded", "Excluded", "Excluded", "Excluded", "Excluded", "Excluded",
"Excluded", "Excluded", "Excluded", "Excluded", "Excluded", "Excluded", "Excluded",
"Excluded", "Excluded", "Excluded", "Excluded", "Excluded", "Excluded", "Excluded",
"Excluded", "Excluded", "Excluded", "Excluded", "Excluded", "Excluded", "Excluded",
"Excluded", "Excluded", "Excluded", "Excluded", "Excluded", "Excluded", "Excluded",
"Excluded", "Excluded", "Excluded", "Excluded", "Excluded", "Excluded", "Excluded",
"Excluded", "Excluded", "Excluded", "Excluded", "Excluded", "Excluded", "Excluded",
"Excluded", "Excluded", "Excluded", "Excluded", "Excluded", "Excluded", "Excluded",
"Excluded", "Excluded", "Excluded", "Excluded", "Excluded", "Excluded", "Excluded",
"Excluded", "Excluded", "Excluded", "Excluded", "Excluded", "Excluded", "Excluded",
"Excluded", "Excluded", "Excluded", "Excluded", "Excluded", "Excluded", "Excluded",
"Excluded", "Excluded", "Excluded", "Excluded", "Excluded", "Excluded", "Excluded",
"Excluded", "Excluded", "Excluded", "Excluded", "Excluded", "Excluded", "Excluded",
"Excluded", "Excluded", "Excluded", "Excluded", "Excluded", "Excluded", "Excluded",
"Excluded", "Excluded", "Excluded", "Excluded", "Excluded", "Excluded", "Excluded",
"Excluded", "Excluded", "Excluded", "Excluded", "Excluded", "Excluded", "Excluded",
"Excluded", "Excluded", "Excluded", "Excluded", "Excluded", "Excluded", "Excluded",
"Excluded", "Excluded", "Included", "Included", "Included", "Included", "Included",
"Included", "Included", "Included", "Included", "Included", "Included", "Included",
"Included", "Included", "Included", "Included", "Included", "Included", "Included",
"Included", "Included", "Included", "Included", "Included", "Included", "Included",
"Included", "Included", "Maybe", "Maybe", "Maybe", "Maybe", "Maybe", "Maybe",
"Maybe", "Maybe", "Maybe", "Maybe", "Included", "Included", "Excluded", "Maybe",
"Excluded", "Included", "Excluded", "Included", "Included", "Excluded", "Included",
"Included", "Included", "Excluded", "Excluded", "Excluded")*

Create a dataframe with reviewer classifications
ratings <- data.frame(FFSC, CPBA)

Compute Cohen's Kappa coefficient
kappa_result <- kappa2(ratings)

Display the result
print(kappa_result)

Output
Cohen's Kappa for 2 Raters (Weights: unweighted)

Subjects = 223

Raters = 2
Kappa = 0.805
z = 14.9
p-value = 0

Full Text Screening

Create the classification matrix based on screening decisions
FFSC <- c("Excluded", "Excluded", "Included", "Excluded", "Excluded", "Excluded", "Excluded", "Excluded", "Excluded", "Excluded", "Included", "Excluded", "Included", "Included", "Included", "Excluded", "Included", "Included", "Excluded", "Excluded", "Excluded", "Included", "Included", "Excluded", "Excluded", "Included", "Included", "Included")
CPBA <- c("Excluded", "Excluded", "Included", "Excluded", "Excluded", "Excluded", "Excluded", "Excluded", "Excluded", "Excluded", "Included", "Excluded", "Included", "Included", "Included", "Included", "Included", "Included", "Excluded", "Excluded", "Excluded", "Included", "Included", "Excluded", "Excluded", "Included", "Included", "Included")

Create a dataframe with reviewer classifications
ratings <- data.frame(FFSC, CPBA)

Compute Cohen's Kappa coefficient
kappa_result <- kappa2(ratings)

Display the result
print(kappa_result)

Output
Cohen's Kappa for 2 Raters (Weights: unweighted)

Subjects = 28
Raters = 2
Kappa = 0.928
z = 4.92
p-value = 8.55e-07
