# Supplementary material for: Mobile and Web Apps for Weight Management in Overweight and Obese Adults: An Updated Umbrella Review and Meta-Analysis
Source: Int J Environ Res Public Health. 2025 Jul 21;22(7):1152. doi: 10.3390/ijerph22071152 (PMC12294216; doi:10.3390/ijerph22071152)
Supplement: Supplementary file 1 [file ijerph-22-01152-s001.zip › File S3. R Scripts for Data Extraction, Subgroup Selection, and Meta-Analyses.docx.pdf]

File S3. R Scripts for Data Extraction, Subgroup Selection, and Meta-Analyses

For full reproducibility, the CSV data file and code are available upon request.

1. Data Import and Preparation

```
library(meta)
library(dplyr)
library(readr)

dados <- read_csv("sum1.csv")
```

2. Meta-Analysis for Each Subgroup

2.1. Mobile App Only (Excluding SMS/Telephone)

```
mobileapp_regex <- "smartphone|mobile app|mobile-based"
exclude_regex <- "SMS|text|telephone|call"

dados_mobileapp <- dados %>%
  filter(grepl(mobileapp_regex, Subgroup, ignore.case=TRUE)) %>%
  filter(!grepl(exclude_regex, Subgroup, ignore.case=TRUE)) %>%
  group_by(`Review Author`) %>%
  arrange(desc(as.numeric(N)), desc(Year)) %>%
  slice(1) %>% ungroup()

if (nrow(dados_mobileapp) >= 2) {
  meta_mobileapp <- metagen(
    TE = as.numeric(dados_mobileapp$`Effect Size (Value)`),
    seTE = as.numeric(dados_mobileapp$SE),
    studlab = dados_mobileapp$`Review Author`,
    data = dados_mobileapp,
    random = TRUE,
    sm = "MD"
  )
  forest(meta_mobileapp, main = "Mobile App Only")
} else {
  print("Less than two studies for meta-analysis (Mobile App Only).")
}
```

2.2. Web-Based Interventions With Human Contact

```
web_contact_regex <-
"web.*contact|coaching|interactive|support|human"

dados_web_contact <- dados %>%
  filter(grepl(web_contact_regex, Subgroup, ignore.case=TRUE)) %>%
  group_by(`Review Author`) %>%
  arrange(desc(as.numeric(N)), desc(Year)) %>%
  slice(1) %>% ungroup()

if (nrow(dados_web_contact) >= 2) {
  meta_web_contact <- metagen(
    TE = as.numeric(dados_web_contact$`Effect Size (Value)`),
    seTE = as.numeric(dados_web_contact$SE),
    studlab = dados_web_contact$`Review Author`,
    data = dados_web_contact,
    random = TRUE,
    sm = "MD"
  )
  forest(meta_web_contact, main = "Web-based with Human Contact")
} else {
  print("Less than two studies for meta-analysis (Web-based Contact).")
}
```

2.3. Web-Based Interventions Without Human Contact

```
web_nocontact_regex <- "web"
exclude_contact_regex <- "web.*contact|coaching|support|human"

dados_web_nocontact <- dados %>%
  filter(grepl(web_nocontact_regex, Subgroup, ignore.case=TRUE))
%>%
  filter(!grepl(exclude_contact_regex, Subgroup, ignore.case=TRUE))
%>%
  group_by(`Review Author`) %>%
  arrange(desc(as.numeric(N)), desc(Year)) %>%
  slice(1) %>% ungroup()

if (nrow(dados_web_nocontact) >= 2) {
  meta_web_nocontact <- metagen(
    TE = as.numeric(dados_web_nocontact$`Effect Size (Value)`),
    seTE = as.numeric(dados_web_nocontact$SE),
    studlab = dados_web_nocontact$`Review Author`,
    data = dados_web_nocontact,
    random = TRUE,
```

```
    sm = "MD"
  )
  forest(meta_web_nocontact, main = "Web-based without Human
Contact")
} else {
  print("Less than two studies for meta-analysis (Web-based No
Contact).")
}
```

2.4. Duratio Subgroup - Short Term ≤6 months)

```
shortterm_regex <- "short|≤06 months|01 month|02 months|03
months|04 months|05 months|06 months"
dados_shortterm <- dados %>%
  filter(grepl(shortterm_regex, Subgroup, ignore.case=TRUE)) %>%
  group_by(`Review Author`) %>%
  arrange(desc(as.numeric(N)), desc(Year)) %>%
  slice(1) %>% ungroup()

if (nrow(dados_shortterm) >= 2) {
  meta_shortterm <- metagen(
    TE = as.numeric(dados_shortterm$`Effect Size (Value)`),
    seTE = as.numeric(dados_shortterm$SE),
    studlab = dados_shortterm$`Review Author`,
    data = dados_shortterm,
    random = TRUE,
    sm = "MD"
  )
  forest(meta_shortterm, main = "Short-term Interventions")
} else {
  print("Less than two studies for meta-analysis (Short-term).")
}
```

2.5. Duration Subgroup - Long Term ≥12 months

```
longterm_regex <-
"(long|≥?\\s*1[2-9]\\s*months?|≥?\\s*[2-9][0-9]\\s*months?|\\d{2,}\\s*mon
ths?|long-term|duration ?= ?1[2-9]|duration ?= ?[2-9][0-9])"
dados_longterm <- dados %>%
  filter(grepl(longterm_regex, Subgroup, ignore.case=TRUE)) %>%
  group_by(`Review Author`) %>%
```

```
  arrange(desc(as.numeric(N)), desc(Year)) %>%
  slice(1) %>%
  ungroup()

if (nrow(dados_longterm) >= 2) {
  meta_longterm <- metagen(
    TE = as.numeric(dados_longterm$`Effect Size (Value)`),
    seTE = as.numeric(dados_longterm$SE),
    studlab = dados_longterm$`Review Author`,
    data = dados_longterm,
    random = TRUE,
    sm = "MD"
  )
  forest(meta_longterm, main = "Long-term Interventions")
} else {
  print("Less than two studies for meta-analysis (Long-term).")
}
```

2.6. Outcome Subgroup - BMI

```
weight_regex <- "(weight|body weight|weight loss|weight change)"
dados_weight <- dados %>%
  filter(grepl(weight_regex, Outcome, ignore.case = TRUE) |
grepl(weight_regex, Subgroup, ignore.case = TRUE)) %>%
  group_by(`Review Author`) %>%
  arrange(desc(as.numeric(N)), desc(Year)) %>%
  slice(1) %>%
  ungroup()

if (nrow(dados_weight) >= 2) {
  meta_weight <- metagen(
    TE = as.numeric(dados_weight$`Effect Size (Value)`),
    seTE = as.numeric(dados_weight$SE),
    studlab = dados_weight$`Review Author`,
    data = dados_weight,
    random = TRUE,
    sm = "MD"
  )
  forest(meta_weight, main = "Forest plot for Weight")
} else {
  print("Less than two studies for meta-analysis (Weight).")
}
```

2.7. Outcome Subgroup - BMI

```
bmi_regex <- "BMI|body mass index"
dados_bmi <- dados %>%
  filter(grepl(bmi_regex, Outcome, ignore.case=TRUE) | grepl(bmi_regex,
Subgroup, ignore.case=TRUE)) %>%
  group_by(`Review Author`) %>%
  arrange(desc(as.numeric(N)), desc(Year)) %>%
  slice(1) %>%
  ungroup()

if (nrow(dados_bmi) >= 2) {
  meta_bmi <- metagen(
    TE = as.numeric(dados_bmi$`Effect Size (Value)`),
    seTE = as.numeric(dados_bmi$SE),
    studlab = dados_bmi$`Review Author`,
    data = dados_bmi,
    random = TRUE,
    sm = "MD"
  )
  forest(meta_bmi, main = "BMI")
} else {
  print("Less than two studies for meta-analysis (BMI).")
}
```

2.8. Outcome Subgroup - Waist Circumference

```
waist_regex <- "waist"
dados_waist <- dados %>%
  filter(grepl(waist_regex, Outcome, ignore.case=TRUE) | grepl(waist_regex,
Subgroup, ignore.case=TRUE)) %>%
  group_by(`Review Author`) %>%
  arrange(desc(as.numeric(N)), desc(Year)) %>%
  slice(1) %>%
  ungroup()

if (nrow(dados_waist) >= 2) {
  meta_waist <- metagen(
    TE = as.numeric(dados_waist$`Effect Size (Value)`),
    seTE = as.numeric(dados_waist$SE),
    studlab = dados_waist$`Review Author`,
    data = dados_waist,
    random = TRUE,
    sm = "MD"
  )
```

```
  forest(meta_waist, main = "Waist Circumference")
} else {
  print("Less than two studies for meta-analysis (Waist Circumference).")
}
```

Single-Study Subgroup Analysis

2.8. Outcome Subgroup - Body Fat Percentage

```
bodyfat_regex <- "body fat|fat %|fat percentage"

bodyfat_sub <- dados %>%
  filter(grepl(bodyfat_regex, Outcome, ignore.case = TRUE) |
         grepl(bodyfat_regex, Subgroup, ignore.case = TRUE)) %>%
  group_by(`Review Author`) %>%
  arrange(desc(as.numeric(N)), desc(Year)) %>%
  slice(1) %>%
  ungroup()

print(paste("Número de estudos para body fat:", nrow(bodyfat_sub)))

  meta_bodyfat <- metagen(
    TE = as.numeric(bodyfat_sub$`Effect Size (Value)`),
    seTE = as.numeric(bodyfat_sub$SE),
    studlab = bodyfat_sub$`Review Author`,
    data = bodyfat_sub,
    sm = "MD"
  )
  forest(meta_bodyfat, main = "Forest plot for Body Fat (%) — Single Study")
}
```
