# Supplementary material for: Mobile and Web Apps for Weight Management in Overweight and Obese Adults: An Updated Umbrella Review and Meta-Analysis
Source: Int J Environ Res Public Health. 2025 Jul 21;22(7):1152. doi: 10.3390/ijerph22071152 (PMC12294216; doi:10.3390/ijerph22071152)
Supplement: Supplementary file 1 [file ijerph-22-01152-s001.zip › Table S2. Reasons for Full-Text Exclusion.docx.pdf]

Table S2. Reasons for Full-Text Exclusion

Title	Year	First Author	Reason for Exclusion
A systematic review of tailored eHealth interventions for weight loss.	2019	RYAN, K.	No meta-analysis conducted
The evolution and effects of mobile health (mHealth) intervention on weight management among healthy overweight/obese populations in China: a systematic review and meta-analysis.	2022	CHEN, M.	Ineligible population (age group not clearly defined or stratified)
Remotely Delivered Interventions for Obesity Treatment.	2019	BRADLEY, L. E.	Narrative review (not systematic)
Can Mobile Technology Improve Weight Loss in Overweight Adults? A Systematic Review.	2020	WANG, E.	No meta-analysis conducted
Use of Mobile Phone App Interventions to Promote Weight Loss: Meta-Analysis.	2020	ISLAM, M. M.	Ineligible population (age group not clearly defined or stratified)
Sustainability of Weight Loss Through Smartphone Apps: Systematic Review and Meta-analysis on Anthropometric, Metabolic, and Dietary Outcomes.	2022	CHEW, H. S. J.	Ineligible population (age group not clearly defined or stratified)
Effectiveness and Components of Web-Based Interventions on Weight Changes in Adults Who Were Overweight and Obese: A Systematic Review with Meta-Analyses.	2022	SHI, Y.	Non-eligible intervention (multicomponent digital strategy; effect of apps not isolated)
Promoting Physical Activity and Weight Loss With mHealth Interventions Among Workers: Systematic Review and Meta-analysis of	2022	JUNG, J.	Non-eligible intervention (apps/web tools not analyzed independently from wearable devices)

Randomized Controlled Trials.			
Associations between behaviour change technique clusters and weight loss outcomes of automated digital interventions: a systematic review and meta-regression.	2023	BERRY, M. P.	Ineligible population (BMI ≥23; no stratification by overweight/obese status)
Mobile Phone Apps to Promote Weight Loss and Increase Physical Activity: A Systematic Review and Meta-Analysis.	2015	FLORES MATEO, G.	Ineligible population (age group not clearly defined or stratified)
Can Digital Technologies Be Useful for Weight Loss in Individuals with Overweight or Obesity? A Systematic Review.	2024	PROTANO, C.	Non-eligible intervention (apps/web tools not analyzed independently from wearable devices)
[Mobile health and excess weight: a systematic review].	2014	SARNO, F.	No meta-analysis conducted
Mobile apps for weight management in children and adolescents; An updated systematic review.	2021	LANGARIZADEH, M.	No meta-analysis conducted
The Influence of Physical Activity and Diet Mobile Apps on Cardiovascular Disease Risk Factors: Meta-Review.	2024	BUSHEY, E.	Umbrella review with ineligible population (focus on cardiovascular risk, no stratification)
Self-Monitoring via Digital Health in Weight Loss Interventions: A Systematic Review Among Adults with Overweight or Obesity.	2021	PATEL, M. L.	No meta-analysis conducted
Assessment of the Efficacy, Safety, and Effectiveness of Weight Control and Obesity Management Mobile Health Interventions: Systematic Review.	2019	PUIGDOMENECH PUIG, E.	No meta-analysis conducted
