# Supplementary material for: Mobile and Web Apps for Weight Management in Overweight and Obese Adults: An Updated Umbrella Review and Meta-Analysis
Source: Int J Environ Res Public Health. 2025 Jul 21;22(7):1152. doi: 10.3390/ijerph22071152 (PMC12294216; doi:10.3390/ijerph22071152)
Supplement: Supplementary file 1 [file ijerph-22-01152-s001.zip › Table S3. Overlap of primary studies.docx.pdf]

**Table S3**. **Overlap of primary studies**

| | Reviews | | | | | | | | | | |
|---|---|---|---|---|---|---|---|---|---|---|---|
| **Primary Studies** | **Cavero-Redondo et al. (2020) [18]** | **Park et al. (2019) [10]** | **Zheng et al. (2023) [19]** | **Khokhar et al. (2014) [20]** | **Varela et al. (2021) [21]** | **Neve et al. (2010) [9]** | **Hutchesson et al. (2015) [22]** | **Metzendorf et al. (2024) [23]** | **Jahangiry & Farhangi (2024) [24]** | **Holm et al. (2024) [25]** | **Beleigoli et al. (2019) [26]** |
| **Allen et al. (2013)** | x | x | | | | | x | | | | x |
| **Allman-Farinelli et. al (2016)** | x | | | | | | | | | | |
| **Burke et al. (2011)** | x | | | | | | | | | | |
| **Carter et al. (2013)** | x | x | | x | | | | x | | | |
| **Hartman et al. (2016)** | x | | | | | | | | | | |
| **Hutchesson et. al (2018)** | x | | | | | | | | | | |
| **Jospe et al. (2017)** | x | | | | | | | | | | |
| **Martin et al. (2015)** | x | x | | | | | | | | | |
| **Morgan et al. (2013)** | x | | | | x | | | | | | |
| **Park et al. (2012)** | x | | | | | | | | | | |
| **Ross et al. (2016)** | x | | | | | | | | | | |
| **Sniehotta et al. (2019)** | x | | | | | | | | | | |

| | | | | |
|---|---|---|---|---|
| Spring et al. (2013) | x | x | x | |
| Stephens et al. (2017) | x | | | x |
| Svetkey et al. (2015) | x | x | | |
| Teerinemi et al. (2018) | x | | | |
| Thomas et al. (2019) | x | | | |
| Wang et al. (2018) | x | | | |
| Wharton et al. (2014) | x | | | |
| Yon et al. (2007) | x | | | |
| Hale et al. (2016) | | x | | |
| Partridge et al. (2016) | | x | | |
| Ross & Wing (2016) | | x | | |
| Sidhu et al. (2016) | | x | | |
| Cadmus-Bertram et al. (2015) | | x | | |
| Laing et al. (2014) | | x | | |
| Lin et al. (2014) | | x | x | |
| Hebden et al. (2013) | | x | | |

| | | | | | |
|---|---|---|---|---|---|
| **Norman et al. (2013)** | **x** | | | | |
| **Shaw et al. (2013)** | **x** | | | **x** | |
| **Steinberg et al. (2013)** | **x** | | | **x** | **x** |
| **Shapiro et al. (2012)** | **x** | | **x** | **x** | |
| **Turner-McGrievy & Tate (2011)** | **x** | | | | |
| **Ahtinen et al. (2013)** | | **x** | | | |
| **Van Drongelen et al. (2014)** | | **x** | | | |
| **Du et al. (2016)** | | **x** | | | |
| **Zhang et al. (2017)** | | **x** | | | |
| **Comulada et al. (2017)** | | **x** | | | |
| **Ashton et al. (2017)** | | **x** | | | |
| **Baek et al. (2018)** | | **x** | | | |
| **Podina et al. (2018)** | | **x** | | | |
| **Gleen et al. (2019)** | | **x** | | | |
| **Jiang et al. (2019)** | | **x** | | | |

| | | | | |
|---|---|---|---|---|
| Mhurchu et al. (2019) | x | | | |
| Oftedal et al. (2019) | x | | | |
| Brindal et al. (2019) | x | | | |
| Lyzwinski et al. (2019) | x | | | |
| Torres et al. (2020) | x | | | |
| Bonn et al. (2022) | x | | | |
| Cantisano et al. (2022) | x | | | |
| Yuan et al. (2022) | x | | | |
| Wilson et al. (2023) | x | | x | |
| Napolitano et al. (2013) | x | x | | |
| Norton et al. (2015) | x | | | |
| Hartin et al. (2016) | x | | | |
| Schiwal et al. (2020) | x | | | |
| Block et al. (2015) | x | | | x |
| Lin et al. (2015) | x | | | |

| | | |
|---|---|---|
| Mattila et al. (2016) | x | |
| Järvelä-Reijonen et al. (2018) | x | |
| Järvelä-Reijonen et al. (2020) | x | |
| Stahl et al. (2020) | x | |
| Puntpanich et al. (2020) | x | |
| Duncan et al. (2020) | x | |
| Fenton et al. (2021) | x | |
| Nezami et al. (2021) | x | |
| Drew et al. (2021) | x | |
| Young et al. (2021) | x | |
| Drew et al. (2022) | x | |
| Napolitano et al. (2021) | x | |
| Didehban et al. (2022) | x | |
| Nakata et al. (2022) | x | x |
| Thorgeirsson et al. (2022) | x | |

| | | | | | | |
|---|---|---|---|---|---|---|
| Jensen et al. (2023) | x | | | | | |
| Beasley et al. (2008) | | x | | | | |
| Haapala et al. (2009) | | x | | | x | |
| Patrick et al. (2009) | | x | | | x | |
| Burke et al. (2012) | | x | | | x | |
| Blomfield et al. (2013) | | | x | | | |
| Chambliss et al. (2011) | | | x | | x | |
| Collins et al. (2012) | | | x | | x | x |
| Collins et al. (2013) | | | x | | | |
| Gabriele et al. (2010) | | | x | | | |
| Gold et al. (2007) | | | x | x | x | |
| Hunter et al. (2008) | | | x | | x | |
| Morgan et al. (2009) | | | x | | | |
| Morgan et al. (2010) | | | x | | | |

| | | | | | | |
|---|---|---|---|---|---|---|
| Morgan et al. (2011) | | x | | x | | |
| O'Brien et al. (2014) | | x | | | | |
| Tate et al. (2001) | | x | x | x | | |
| Thomas et al. (2017) | | x | | | | |
| Womble et al. (2004) | | x | x | x | | |
| Carr et al. (2008) | | | x | | | |
| Cussler et al. (2008) | | | x | x | | |
| McConnon et al. (2007) | | | x | x | | x |
| Micco et al. (2007) | | | x | x | | |
| Mobley (2006) | | | x | | | |
| Polzien et al. (2007) | | | x | | | |
| Rothert et al. (2006) | | | x | x | | |
| Tate et al. (2006) | | | x | x | | |
| Tate et al. (2003) | | | x | x | | |
| Webber (2007) | | | x | | | |
| Harvey-Berino et al. (2002) | | | x | x | | |

| | | |
|---|---|---|
| Harvey-Berino et al. (2004) | x | x |
| Svetkey et al. (2008) | x | x |
| Ackermann et al. (2014) | | x |
| Appel et al. (2011) | | x |
| Bennett et al. (2012) | | x |
| Bennett et al. (2010) | | x |
| Booth et al. (2008) | | x |
| Brindal et al. (2012) | | x |
| Cadmus-Bertram et al. (2013) | | x |
| Carnie et al. (2014) | | x |
| Christian et al. (2011) | | x |
| Colleran et al. (2012) | | x |
| Digenio et al. (2009) | | x |
| Gabrielle et al. (2011) | | x |

| | | | |
|---|---|---|---|
| Greene et al. (2013) | x | | |
| Goulis et al. (2004) | x | | |
| Haddock et al. (2014) | x | | |
| Harvey-Berino et al. (2010) | x | | |
| Hersey et al. (2012) | x | | |
| Imanaka et al. (2013) | x | | |
| Johnston et al. (2013) | x | | |
| Kraschnewski et al. (2011) | x | | x |
| Luley et al. (2011) | x | | |
| Leahey et al. (2014) | x | | |
| McDoniel et al. (2010) | x | | |
| Mouttappa et al. (2011) | x | | |
| Patrick et al. (2011) | x | | |
| Pellegrini et al. (2012) | x | x | |

| | | |
|---|---|---|
| Polzein et al. (2007) | x | |
| Pullen et al. (2008) | x | |
| Schroeder et al. (2010) | x | |
| Shrestha et al. (2013) | x | |
| Shuger et al. (2011) | x | |
| Turner-McGrievy et al. (2011) | x | |
| Turner et al. (2009) | x | |
| Van Wier et al. (2011) | x | |
| Webber et al. (2013) | x | |
| Webber et al. (2010) | x | |
| Webber et al. (2008) | x | |
| Wing et al. (2010) | x | |
| Wylie Roset et al. (2001) | x | |
| Yardley et al. (2014) | x | x |

| | | |
|---|---|---|
| Gerber et al. (2013) | x | |
| Thomas et al. (2011) | x | |
| Thorndike et al. (2012) | x | |
| Wing et al. (2006) | x | |
| Sullivan et al. (2013) | x | |
| Bennett et al. (2013) | x | |
| Gow et al. (2010) | x | |
| Hebden et al. (2014) | x | |
| Kelders et al. (2011) | x | |
| Lachausse et al. (2012) | x | |
| Lombard et al. (2010) | x | |
| Van et al. (2012) | x | |
| Winett et al. (2011) | x | |
| Apiñaniz et al. (2019) | | x |
| Batch et al. (2014) | | x |

| | | | |
|---|---|---|---|
| Castellano-Tejedor et al. (2017) | x | | |
| Eisenhauer et al. (2020) | x | | |
| EVIDENT3 Group (2022) | x | | |
| Hurkmans et al. (2018) | x | | x |
| Joseph et al. (2019) | x | | |
| Kliemann et al. (2019) | x | | |
| L'Allemand et al. (2018) | x | | |
| Locke et al. (2020) | x | | |
| Roth et al. (2023) | x | | |
| Tanaka et al. (2018) | x | | |
| Van Beurden et al. (2019) | x | | |
| Vidmar et al. (2019) | x | | |
| McDoniel et al (2010) | | x | |
| Shaunger et al. (2011) | | x | |

| | | |
|---|---|---|
| Hutchesson et al. (2014) | x | |
| Mehring et al. (2013) | x | |
| Carr et al. (2009) | x | |
| Park MJ et al. (2012) | x | |
| Alfawaz et al. (2019) | | x |
| Alfawaz et al. (2018) | | x |
| Al-Hamdan et al. (2021) | | x |
| Bhopal et al. (2014) | | x |
| Block et al. (2016) | | x |
| Bootwong & Intarut (2022) | | x |
| Chattopadhyay et al. (2023) | | x |
| Chung et al. (2023) | | x |
| Davies et al. (2016) | | x |
| Dawes et al. (2015) | | x |

| | |
|---|---|
| Fischer et al. (2016) | x |
| Fottrell et al. (2023) | x |
| Griauzde et al. (2019) | x |
| Han et al. (2024) | x |
| Katula et al. (2022) | x |
| Kitazawa et al. (2023) | x |
| Lee et al. (2021) | x |
| Lim et al. (2022) | x |
| Mann, Palmisano & Lin (2016) | x |
| Muilwijk et al. (2021) | x |
| Nanditha et al. (2018) | x |
| O’Brien et al. (2017) | x |
| Saboo, Kacker & Rathore (2021) | x |
| Sakane et al. (2011) | x |
| Sampson et al. (2021) | x |

| | | |
|---|---|---|
| Toro-Ramos et al. (2020) | x | |
| Wani et al. (2020) | x | |
| Wein et al. (1999) | x | |
| Wong et al. (2013) | x | |
| Wong et al. (2018) | x | |
| Chung et al. (2014) | | x |
| Dunn et al. (2016) | | x |
| Krukowski et al. (2011) | | x |
| Padwal et al. (2017) | | x |
